# The complete mitochondrial genome of *Solemya velum* (Mollusca: Bivalvia) and its relationships with Conchifera

**DOI:** 10.1186/1471-2164-14-409

**Published:** 2013-06-18

**Authors:** Federico Plazzi, Anisa Ribani, Marco Passamonti

**Affiliations:** 1 Department of Biological Geological and Environmental Sciences, University of Bologna, Via Selmi, 3, Bologna 40126, Italy

**Keywords:** Solemya velum, Mitochondrial genome, Gene arrangement, Origin of replication, Mitogenomics

## Abstract

**Background:**

Bivalve mitochondrial genomes exhibit a wide array of uncommon features, like extensive gene rearrangements, large sizes, and unusual ways of inheritance. Species pertaining to the order Solemyida (subclass Opponobranchia) show many peculiar evolutionary adaptations, f.i. extensive symbiosis with chemoautotrophic bacteria. Despite Opponobranchia are central in bivalve phylogeny, being considered the sister group of all Autobranchia, a complete mitochondrial genome has not been sequenced yet.

**Results:**

In this paper, we characterized the complete mitochondrial genome of the Atlantic awning clam *Solemya velum*: A-T content, gene arrangement and other features are more similar to putative ancestral mollusks than to other bivalves. Two supranumerary open reading frames are present in a large, otherwise unassigned, region, while the origin of replication could be located in a region upstream to the *cox3* gene.

**Conclusions:**

We show that *S. velum* mitogenome retains most of the ancestral conchiferan features, which is unusual among bivalve mollusks, and we discuss main peculiarities of this first example of an organellar genome coming from the subclass Opponobranchia. Mitochondrial genomes of *Solemya* (for bivalves) and *Haliotis* (for gastropods) seem to retain the original condition of mollusks, as most probably exemplified by *Katharina*.

## Background

### Bivalves and mitochondrial DNA

In animals, the mitochondrial genome (mtDNA) is typically a small, circular and compact molecule, generally encoding for 37 genes: 13 protein-coding genes (PCGs), 2 rRNAs, and 22 tRNAs [1-3]. Even if striking exceptions to this standard are known [4-10], most differences among animal mtDNAs involve gene content and arrangement.

Mollusks have shown high variability in mitochondrial genome architecture [3,11-13], with respect to many genomic features, *i.e.* length, gene arrangement, strand assignment, gene duplications and losses, nucleotide composition, and more. Within mollusks, gastropods and bivalves show extensive variations, even with differences within the same family or genus [11,14,15]. Furthermore, a major peculiar trait of mitochondrial genome in some bivalve species is the presence of an alternative pattern of mitochondrial inheritance known as Doubly Uniparental Inheritance or DUI [16-20], that involves two separate lineages of mtDNAs. One mitochondrial genome (called F) is transmitted from the mother to the complete offspring, whereas the other one (called M) is transmitted from the father to sons, where it localizes in germline and, therefore, in sperm. Still, differences in gene order were also detected between the two DUI-related lineages within the same species [13,21,22].

Mitochondrial gene order has been shown to be a good marker for phylogenetic relationships, because rearrangements are random discrete events, and retro-mutation is very unlikely (see, f.i., [23-26]; and reference therein). This is even more true for arrangements of protein coding or rRNA genes, because they are much rarer than the ones involving tRNAs [1,11,13,27,28].

Tracing the “archaic” mtDNA gene arrangement of Mollusca, we can start from groups sequenced so far seeming to retain most ancestral features. Among them, chitons are aculiferan mollusks that are considered sister group to all conchiferans, which include scaphopods, cephalopods, gastropods, and bivalves (see, f.i., [29-31]; and reference therein). For PCGs and rRNAs, the gene arrangement of the chiton *Katharina tunicata*[32] is also shared by *Haliotis*, a primitive gastropod [33]; a single inversion separates it from the gene arrangement of Caenogastropoda (with the exception of *D. gregarium*; see [15,34]); few steps are required to transform the gene order of *Katharina* in that of *Nautilus*, a primitive cephalopod [35]. A single translocation also separates it from the aplacophoran *C. nitidulum* ([GenBank:EF211990]). Moreover, the gene arrangement of *Katharina tunicata* shows some outstanding similarities to lophophorates and even arthropods [27,32,36]. This clearly points out that *Katharina* may have the most “archaic” gene order known so far among Mollusca, and maybe the ancestral mollusk gene arrangement [13,28].

Many exceptions to typical gene content are known: f.i., it is well known that the *atp8* gene has been reported as missing in several bivalve species, as discussed in ([37]; and reference therein). *atp8* is present on the same strand in all Unionoida ([37], and reference therein); as a single exception, it is incomplete in the male mtDNA of *Pyganodon grandis*[22,38]. Moreover, it has been found in some heterodonts, like *Loripes lacteus* ([GenBank:EF043341]), *Lucinella divaricata* ([GenBank:EF043342]), *Meretrix lamarckii*[39], and *Meretrix lusoria*[40]; a putative *atp8* has also been recently reported from the mytilid *Musculista senhousia*[18]. Conversely, it was not recovered in the mactrid *Coelomactra antiquata*[41].

Among the other exceptions to gene features and content, the *rrnS* gene is duplicated in some species of genus *Crassostrea*, while the *rrnL* gene is split in two separate fragments in all ostreids known to date ([14,42-44]; [GenBank:FJ841968]); finally, two versions of *cox2* were found in the *Musculista senhousia* M mtDNA [18].

### The taxonomic position of *Solemya*

Despite sharing a common bivalve shell, two major kind of bivalvian mollusks are known. The first group, the Opponobranchia, including most of the protobranchiate bivalves, is generally deemed to retain many ancestral features, while the second, the Autobranchia, *i.e.* lamellibranch bivalves with completely functional filter-feeding gills, is a big assemblage of species showing a more derived morphology, when compared to putative ancestral mollusks. Among bivalves, complete mitochondrial genomes are nowadays available for Autobranchia only, which show many differences with respect to the condition of *K. tunicata*.

In the subclass Opponobranchia there are basically two groups of living bivalves: Nuculida and Solemyida. The genus *Solemya* and relatives have edentulous or nearly edentulous, generally equivalved shells, with an homogeneous aragonitic ostracum, a glossy, thick and brown periostracum, and the mollusk shows a broad fringe, siphonate mantle and burrowing habits [45,46]. Most solemyids are involved in symbiosis with chemoautotrophic, gill-hosted, bacteria, enabling life in unusual habitats like deep-sea vents [46,47].

In this paper, we present the first complete mitochondrial genome of a representative of Opponobranchia, the Atlantic awning clam *Solemya velum* Say, 1822. This organelle genome was completely annotated and compared to other available bivalve and conchiferan complete mitochondrial genomes. Because of the sister-group relationship between Opponobranchia and Autobranchia, the characteristics of the *Solemya* mitogenome have proved useful to compare bivalve mtDNA features with those of other mollusks. In this paper we discuss our findings with particular reference to: (i) the presence of the *atp8* gene; (ii) nucleotide composition; (iii) strand patterns; (iv) origin of replication; (v) supranumerary ORFs; (vi) gene arrangement.

## Methods

### DNA extraction, Long-PCR reactions and sequencing

Specimens of *Solemya velum* were collected by and commercially purchased at the Woods Hole Oceanographic Institution (Massachusetts, USA) in Summer 2003. A standard phenol:chloroform protocol was used to extract total genomic DNA from a pool of 5 individuals, because of the small dimensions of the obtained specimens.

The technique of Long-PCR amplification, paired with sequencing with primer-walking or shotgun cloning, was used to amplify the complete mitochondrial genome in several overlapping fragments. The Herculase® II Fusion Enzyme (Stratagene) kit was used to perform Long-PCR reactions up to 10,000 bp. The reaction conditions were set as follows: 10 μL 5× Herculase® II Fusion reaction buffer, nucleotides 250 μM each, primers 0.25 μM each, 0.5 μL Herculase® II Fusion, 5 μL template DNA, ddH_2_O up to 50 μL. Cycle conditions were set up as an initial denaturation step at 92°C for 2′, 40 cycles of denaturation at 92°C for 10′, annealing at 48-52°C for 30'', and extension at 68°C for 10', and a final extension step of 68°C for 8′. Primers used for Long-PCR were used to sequence long amplicons and new internal specific primers to complete primer-walking were designed with Primer3 online tool [48].

Routine PCR amplification was performed for amplicons <2,000 bp with GoTaq® Flexi DNA Polymerase (Promega) as in [49]. Amplicons were purified through PEG precipitation [50], or with Wizard® SV Gel and PCR Clean-Up System (Promega); when necessary, they were ligated into a pGEM® T-Easy Vector (Promega) and transformed into MAX Efficiency® DH5α™ Chemically Competent Cells (Invitrogen) as in [51]. Additional files 1 and 2 list all primers used for this study. All sequencing reactions were carried out through the Macrogen Europe (Amsterdam, The Netherland) facility. Only in the case of long-PCR with primers COI2F and Solemya_3a1894R (see Additional file 1), the 10,000 bp long amplicon was purified with 5% PEG precipitation [50] and sequenced via shotgun cloning. This was carried out by Macrogen Korea (Seoul, South Korea).

### Sequence annotation

Protein-coding genes were annotated using the online ORF Finder tool [52]; the software Glimmer 3.02 [53] under iterated pipeline for assessing ORF features was used to confirm results; homology search was carried out with BLAST ([54,55]; and reference therein]). We investigated structures and putative functions of unknown ORFs through the @TOME 2.0 ([56]; and reference therein) and InterProScan ([57]; and reference therein) online tools: signal peptides were sought with SignalP [58], while similarities were detected using HHsearch [59], SP^3^[60] and Fugue [61].

Start codons of PCGs were set at the first start codon found by ORF Finder that did not overlap with an upstream gene; whenever a stop codon was overlapping with a following gene, it was moved backwards to the first suitable codon starting with T/TA (thus annotating a hypothetical truncated T--/TA- stop codon). In cases of neighboring PCGs, these *in silico* predictions were tested looking for a secondary structure with a possible cleavage signaling function in the connecting region, using the Mfold server [62] and a folding temperature of 14°C. tRNA genes were predicted with tRNAscan-SE 1.21 [63,64] and ARWEN 1.2 [65]. The Mfold server was used to predict the secondary structure of unassigned regions; all secondary structures were graphically edited with VARNA 3.7 [66].

Codon usage and nucleotide composition statistics were computed using MEGA 5.03 [67] and Microsoft Excel® 2007; repeated sequences were found with Spectral Repeat Finder v 1.1 [68]. The mitochondrial genome map was prepared using GenomeVx [69], setting *cox1* as the starting point of the mtDNA and labeling its coding strand as “ + ”.

### Phylogenetic analysis

Complete mitochondrial genomes of bivalves and other mollusks were downloaded from GenBank in November 2011 (Additional file 3). Summarizing, we included in our dataset 30 bivalves, 23 gastropods, 6 cephalopods, 1 scaphopod, 1 polyplacophoran, 1 chaetodermomorph, and the polychaete outgroup *Platynereis dumerilii*[70]. We assessed phylogenetic representativeness of this sample through the AvTD method as in [49]. We used the software PhyRe [71] and set the number of splits, merges, and moves to 2, shuffling at the family level. Sequences were managed through CLC Sequence Viewer 6.6.2 (CLC bio A/S), Microsoft Excel® 2007, and MEGA 5.03.

Each gene, with the exception of *atp8*, was separately translated into amminoacids and aligned with MAFFT 6 [72] and Muscle 3.8.31 [73,74], using the M-Coffee merging algorithm [75,76]. Gblocks [77,78] was used to select blocks of conserved positions suitable for phylogenetic analysis under default (stringent) conditions.

PartitionFinderProtein 1.0.1 [79], using the greedy option and Bayesian Information Criterion (BIC), tested the best partitioning scheme of our dataset, which was chosen for subsequent analysis, as well as the concatenated alignment and the completely partitioned model. Best-fitting amminoacid substitutions models were selected with ProtTest 3.2 ([80]; and reference therein), through Phyml [81] and BIC for model selection.

The software RAxML 7.2.8 [82,83] was used for maximum likelihood analyses, using both the fast (−x) and the standard (−b) bootstrap algorithm with 200 replicates. The PROTCAT model [84] was implemented for optimization of individual per-site substitution rates, using models suggested by ProtTest 3.2. Trees were graphically edited by PhyloWidget [85], Dendroscope [86], and Inkscape softwares.

## Results

### Genomic features

**Figure 1:**
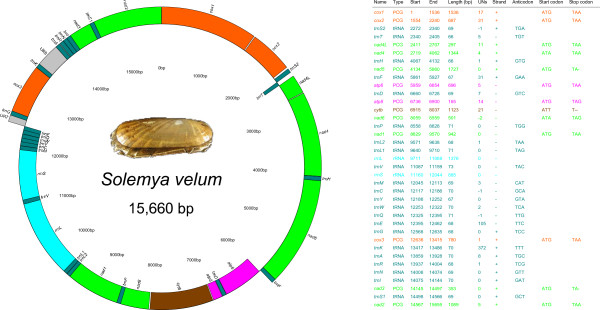
**Genomic map and annotation of *S. velum *complete mitochondrial genome.** Genes located on the external side of the map are on “ + ” strand (*i.e.* that encoding *cox1*), whereas genes on the internal side are on “-” strand. URs > 100 bp are shown and they were arbitrarily set on “ + ” strand. UNs, number of unassigned nucleotides after the gene: if negative, overlapping nucleotides with the following gene; PCG, Protein Coding Gene.

The complete mitochondrial genome of *Solemya velum* was found to be 15,660 bp long. It was deposited into GenBank database under Accession Number [GenBank:NC_017612]. All genes of the standard metazoan mitochondrial genome were found, including the *atp8* gene (Figure 1). With the only exception of *trnT*, genes are organized in a large cluster on the “ + ” strand (from *trnG* to *trnF*) and in a slightly shorter cluster on the “-” strand (from *trnE* to *atp6*). 22 tRNAs are present: as usual for animal mtDNA, two serine-encoding tRNAs, *trnS1(AGN)* and *trnS2(UCN)*, and two leucine-encoding tRNAs, *trnL1(CUN)* and *trnL2(UUR)* were found. The secondary structure of tRNA genes was predicted and is shown in Additional file 4; as expected [1,18,87-90], *trnS1(AGN)* presents a reduced DHU arm.

Nucleotide composition and A-T/G-C proportions were computed for each single gene and for PCGs, third codon positions, ribosomal genes, tRNAs, and URs taken as a whole (Additional file 5): the total A-T content of *S. velum* mitochondrial genome is 68.11%. A chi-square test with 1 d. f. demonstrated that the A-T composition of *S. velum* mtDNA is significantly different from that of other bivalves, gastropods, scaphopods (p < 0.005), and *K. tunicata* (Polyplacophora; p < 0.010); however, no significant difference was found with mtDNA A-T composition of *C. nitidulum* (Caudofoveata) and Cephalopoda (see raw data in Additional file 6).

**Figure 2:**
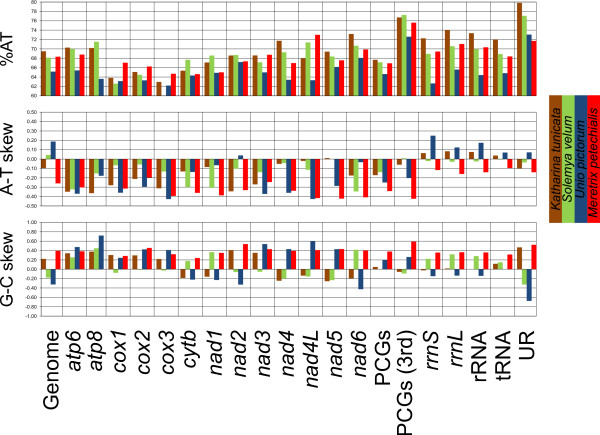
**Compositional patterns of *S. velum *and other mollusks mitochondrial genomes.** A-T content, A-T skew, and G-C skew are computed for each single gene and for many genomic regions following the legend below the chart.

**Figure 3:**
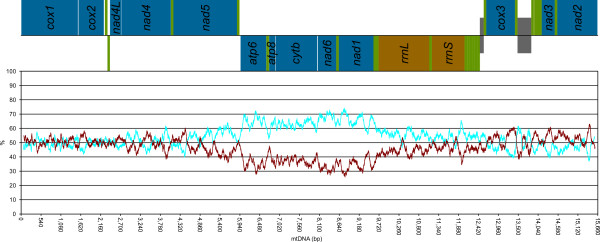
**A-C and G-T content along the mitochondrial genome of *S. velum.*** A-C (pale blue) and G-T (dark red) contents are computed on a sliding window of size 151 bp. A linear sketch of the complete genome as annotated in Figure 1 is depicted above the plot: blue, PCGs; green, tRNAs; brown, rRNAs; gray, URs >100 bp; genes on the “ + ” strand are above the black line and genes on the “-” strand are below it.

A-T content and A-T/G-C skew are shown in Figure 2 for *S. velum* and three other mollusks for comparison: *Katharina tunicata* (Polyplacophora), *Unio pictorum* (Bivalvia: Palaeoheterodonta), and *Meretrix petechialis* (Bivalvia: Heterodonta). A-T content is often similar to that of *K. tunicata* (and *M. petechialis*), while skews, at the genomic level, are more similar to those of *U. pictorum*. We also plotted the A-C and the G-T content along the mitochondrial genome (using the “ + ” strand) with a sliding window of 151 bp (Figure 3). Overall, there is bias towards neither pair, but we evidenced a region of high A-C content spanning approximately from the *atp6* to the *rrnL* gene, *i.e.* part of the mitochondrial genome where all genes are on the “-” strand.

The most common start codon (Figure 1) is ATG (10 PCGs), but also alternative codons were detected, in accordance with previous findings in different invertebrates [1,91]. Most probably, truncated stop codon are used in three genes, namely *nad3*/*nad5* (TA-) and *cytb* (T--). As already shown (f.i., [18,91,92]), these are common in metazoan mitochondrial genomes, with TAA stop codon subsequently restored by post-transcriptional polyadenilation. In five cases (Figure 1), two PCGs are not separated by any tRNA and are neighboring: in all cases, a stem-loop structure with a putative cleavage function of the polycistronic primary transcript has been found (Additional file 7). *S. velum* mtDNA contains 3,735 codifying codons, whose usage is shown in Additional file 8. Most used codon is UUA (Leu), while less used codon is CGC (Arg). The commonest amminoacid is leucine, while the rarest is cysteine.

The third base of the codon is most often an A (38.93%) or a T (38.32%), whereas C (12.39%) and G (10.36%) are less represented in this position. This may simply relate to the degeneracy of the mitochondrial code, but it is possible that natural selection is even looser in these positions because of a wobble effect: as already reported for other metazoans ([18]; and reference therein), only some codons in the PCGs do have their relative tRNA/anticodon on the mtDNA (see Additional file 8).

**Figure 4:**
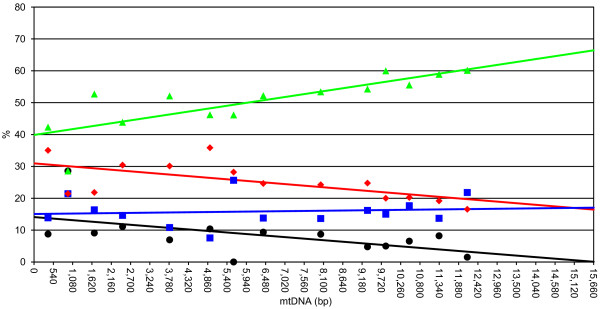
**Location of the origin of replication of the H strand.** A (green), C (blue), G (black), and T (red) content at four-fold degenerate codons of PGCs are shown. Percent contents of each PCG are plotted at the midpoint of the ORF using the first nucleotide of *cox3* ORF as the starting point. We also included ORF117 in this analysis (see text for further details). Equations are as follows. %A, *y* = 0.0017 × + 39.88, r^2^ = 0.64, p < 0.005;%C, *y* = 0.0001 × 15.07, r^2^ = 0.01, p = 0.71;%G, *y* = − 0.0009 × 14.09, r^2^ = 0.30, p < 0.05;%T, *y* = − 0.0009 × 30.96, r^2^ = 0.39, p < 0.05.

We found precise patterns of nucleotide content in four-fold degenerate third codon position along the mtDNA molecule (Figure 4). We used all the PCGs as a possible starting point for the analysis and best results were obtained when *cox3* was used as the first gene in the analysis. All the correlations were found to be significant with the exception of C content (see caption to Figure 4 for details).

### Unassigned regions

Relatively few unassigned regions (URs) are present in the mitochondrial genome of *S. velum* (4.12% of the genome length; Figure 1; Additional files 5 and 9). Most of them are between 11 and 31 bp, but the largest ones are UR7 (105 bp) and UR8 (372 bp), between the *trnE*/*trnG* and *trnK*/*trnA* gene pairs, respectively.

**Figure 5:**
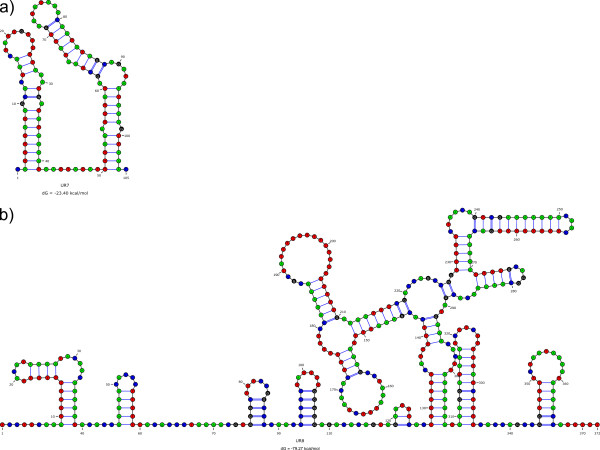
**UR secondary structures.** Putative secondary structures of largest URs of *S. velum* mitochondrial genome were inferred by software Mfold. The Gibbs energy (dG) is shown at the bottom of each structure. **a**, UR7; **b**, UR8.

The putative secondary structures of UR7 and UR8 are shown in Figure 5. UR7 folds as a double hairpin; UR8 folds in a more complex pattern, with several stem-and-loop substructures. A repeated 17-bp long motif was found in this region (5′-ACCAGCCGGTTTTTCTA-3′), starting at bases 220 and 337 of UR8 sequence. Both UR7 and UR8 have a high A-T content (84.76% and 70.43%, respectively), making of UR7 the A-T-richest region in the genome.

Two small ORFs of 117 and 195 nucleotides were found within UR8 and were called ORF117 and ORF195, respectively. ORF117 starts at nucleotide 13,524 (start codon: ATA) and ends at 13,640 (stop codon: TAA) on the “ + ” strand; according to Glimmer results, ORF117 has a “raw” scoring value of 3.05, which is comparable with that of other PCGs, like *nad4L* and *nad6* (2.58 and 3.19, respectively). A BLAST search could find a possible homolog within the putative control region of *Haliotis rubra*[33], which was also confirmed by Glimmer. The A-T content of ORF117 is 59.83% and the most used codon is UUU (Phe), with 7 hits.

ORF195 starts at nucleotide 13,846 (start codon: ATT) and ends at 13,652 (stop codon: TAG) on the “-” strand. ORF195 was not confirmed by Glimmer, but SignalP could retrieve a weak similarity with a signal peptide in the first 29 amminoacids of the putative translated protein; the A-T content of ORF195 is 73.33% and the most used codon is AAA (Lys), with 12 hits.

InterProScan with TMHMM 2.0 online tool could not identify any domain within ORF117, while a transmembrane domain was found within ORF195 (amminoacids 15–32). Using the @TOME 2.0 server, HHsearch, SP^3^ and Fugue could find some similarities between ORF117 and the DNA-binding domains of some proteins ([PDB:1TNS], [PDB:1C20], [PDB:3GNA], [PDB:1G4D]); HHsearch and Fugue also showed homology of ORF195 with two membrane-linked proteins ([PDB:1FME], [PDB:1MEQ]).

### Phylogenetic analysis

The Phylogenetic Representativeness of our sample of mollusk mitogenomes is shown in Additional file 10. Using the original mollusk master list as published by [93], the AvTD of our sample is lying exactly on the mean AvTD of 100 random subsamples of equal size (Additional file 10a, diamond) and it is above the highest random result (Additional file 10b, diamond) if a change in underlying taxonomy is simulated.

The overall length of concatenated alignment, after Gblocks masking, was of 1,782 amminoacids and the *nad4L* gene was completely excluded from the analysis, lacking suitable blocks. The software PartitionFinderProtein selected a 3-blocks model: the first cluster was *atp6*-*cytb*-*nad2*-*nad3*-*nad4*-*nad5*; the second one was *cox1*-*cox2*-*cox3*-*nad1*; the *nad6* gene was given its own partition. All models selected by ProtTest and piped to RAxML are listed in Additional file 11.

**Figure 6:**
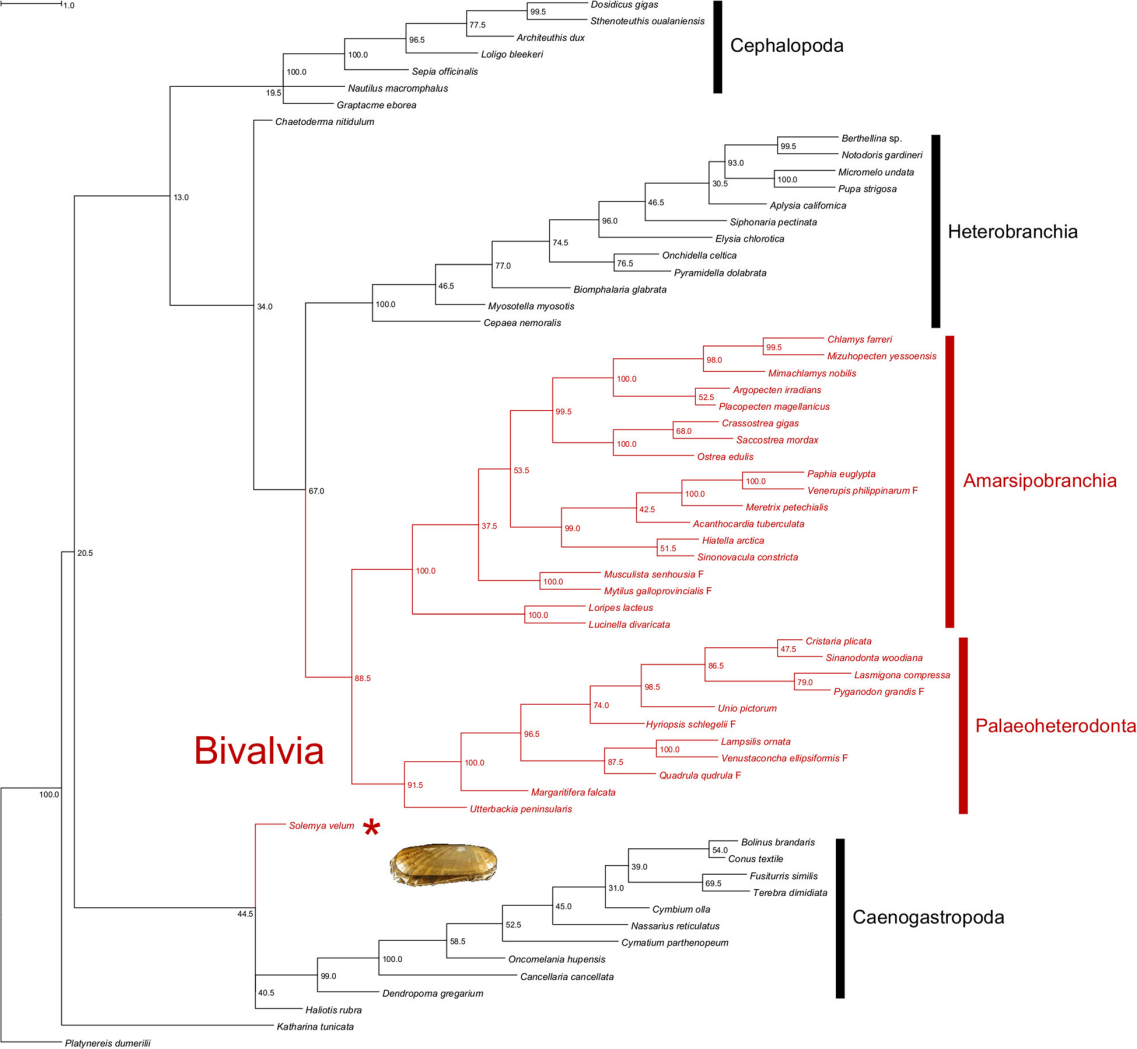
**Maximum likelihood (ML) tree.** The tree shown is the consensus tree of 200 standard bootstrap replicates and the bootstrap proportion (BP) is shown for each node. Bivalves are shown in red; the asterisk indicates *Solemya velum*.

The six ML searches converged on similar trees: following the partitioning scheme selected by PartitionFinderProtein and using the complete bootstrap procedure we obtained the tree shown in Figure 6. *Katharina tunicata* is the sister taxon of all other mollusks; a node with low bootstrap support (BS = 20.5) separates *Solemya* + (*Haliotis* + Caenogastropoda) from (*Graptacme* + Cephalopoda) + (Heterobranchia + Autobranchia). In this scenario, both Bivalvia and Gastropoda seem polyphyletic, but deep nodes are weakly supported (13.0 < BS < 44.5). Conversely, Cephalopoda, Heterobranchia, Amarsipobranchia *sensu*[49], Palaeoheterodonta, and Caenogastropoda were recovered as monophyletic with high BS.

### Gene order

**Figure 7:**
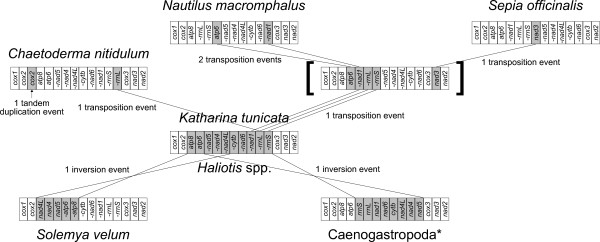
**Gene rearrangements.** Reconstruction of relationships among gene arrangements of *Katharina tunicata*/*Haliotis rubra*/*Haliotis tuberculata*, *Solemya velum*, *C. nitidulum*, Caenogastropoda, *N. macromphalus*, and *S. officinalis* is shown, after the exclusion of tRNAs. Genes involved in the rearrangements are shaded in gray. If a minus sign (“-”) is present, the gene is encoded on the “-” strand, otherwise it is encoded on the “ + ” strand. The asterisk is to signal that *D. gregarium* is an exception to the displayed common gene arrangement of Caenogastropoda; square brackets refer to a possible gene arrangement ancestral to *N. macromphalus* (subclass Nautiloida) and *S. officinalis*.

As expected from the high plasticity of molluscan (and particularly bivalvian) mitochondrial genomes, many differences were found between *S. velum* and other mollusks’ gene arrangement. However, we found that *Katharina tunicata* and *Haliotis rubra* (Gastropoda: Vetigastropoda) share the same gene order (recall that we excluded tRNAs for this analysis) and, more interestingly, that this gene order is highly similar to that of *S. velum*, the only difference being an event of inversion of the *atp8*-*atp6*-*nad5*-*nad4*-*nad4L* cluster (Figure 7). If tRNAs were taken into account, this inversion would hold true (even if the cluster is slightly different due to *trnT*, *trnH*, *trnF*, and *trnD*). The same inversion event can be partially recovered in the unpublished partial mitochondrial genome of the opponobranchiate *Nucula nucleus* (Bivalvia: Nuculida), which is available in GenBank under the Accession Number [GenBank:EF211991].

## Discussion

### Gene content

The *Solemya* mtDNA contains all genes of the standard metazoan mitochondrial genome (Figure 1). It is tempting to conclude that the loss/degeneracy of *atp8* is restricted to Amarsipobranchia, given the presence of this gene in palaeoheterodonts and in *S. velum* (and, following the GenBank partial mitochondrial genome, also in *Nucula nucleus*). Whether the absence of *atp8* gene is real or just an outcome of incorrect annotations (see, f.i., [18,37]), its presence in a bivalve like *Solemya* further supports, if necessary, that the ancestral bivalve condition is the retention of a fully functional *atp8* gene.

Moreover, it is common in metazoans to find neighboring *atp6* and *atp8* on the same strand [1] and it has been suggested that uncleaved transcripts may be co-translated [94,95]. Tough this arrangement is not found in many phyla, like Plathyhelminthes, Nematoda, Annelida, Sipunculida, Brachiopoda, and Mollusca (and *atp8* itself is also lacking in some of them; [13]), nevertheless *atp6* and *atp8* are neighboring in *S. velum* (Tab 3). The same association can be found in basal mollusks, like *C. nitidulum* and *K. tunicata*, and in other conchiferans, like cephalopods (with the exception of *N. macromphalus*), Caenogastropoda, and Heterobranchia (albeit on the opposite strand).

### Genome features

Mean A-T content in main molluscan classes ranges between 63.51% (bivalves) and 74.12% (scaphopod *G. eborea*): *S. velum* has a high A-T content (68.11%), being significantly more similar to aculiferans and cephalopods than to other bivalves (Additional file 6): actually, the A-T content of Autobranchia is between 55.20% (*M. yessoensis*; [96]) and 69.70% (*V. philippinarum*, [GenBank:NC_003354]). Irrespective of the functional constraints and gene features, we found an unbiased, when not low, G-T content in the “ + ” strand (Figure 3). Most commonly, the leading strand is G-T rich ([35,97-103]; but see [104-106]). The G-T content is not particularly high in any region of the *S. velum* “ + ” strand, and it even drops to very low values where the molecule encodes PCGs on the “-” strand (Figure 3).

All genes are located on the same strand in Amarsipobranchia, and most of them in Palaeoheterodonta. Contrastingly, in *Solemya velum*, genes are evenly distributed among “ + ” and “-” strands, with 18 and 19 genes, respectively. Even if a H-biased distribution of genes is found in other lophotrochozoans, like annelids, brachiopods, bryozoans and platyhelminths (see, f.i., [13,27]), an even distribution is the commonest situation among Mollusca (Additional file 6; but see [15]) and, notably, as for A-T content, *S. velum* is quite similar to Caudofoveata, Cephalopoda, Polyplacophora, and Scaphopoda. This gene distribution on both strands rises a stimulating question on strand assignment: which is the leading (heavy; antisense) strand in *S. velum*? Patterns evidenced in *S. velum* resemble those of *N. macromphalus*[35]. Contrarily, a strand with a sharper G-T predominance has been signaled, f.i., in some gastropods [15] and in *Katharina*[35]. It seems that mtDNAs with most genes on the same strand (e.g., Caenogastropoda, Amarsipobranchia) tend to have higher G-T values than mtDNAs with genes evenly distributed on either strand (e.g., Cephalopoda, Palaeoheterodonta).

### Control region and origins of replication

The animal mtDNA control region (CR) should contain or neighbor the origins of replication (ORs). [107] and, specifically for bivalves, Breton and colleagues ([22]; and reference therein) proposed several parameters to annotate the CR, like (i) UR length, (ii) evidence for secondary structure with T-rich loops, (iii) high A-T content and (iv) repetitive elements and palindromes. The best candidates for *S. velum* control region are the unassigned regions UR7 and UR8 (see Additional file 9), but, as expected, they give no BLAST hits with putative CRs of other mollusks. Among the two largest URs, UR8 is the longest unassigned region. Both URs evidenced complex putative secondary structures (Figure 5); again, both URs have a high A-T content, but while the A-T content of UR8 is 70.43% (somewhat near the overall genome score of 68.11%), it is up to 84.76% for UR7, much more than other putative CRs of mollusks [108]. On the other side, the only 17 bp-long repeated motif found in these URs was found in UR8. So, based on the above mentioned characteristics, it is not possible to unambiguously assign the CR function to either UR.

[102] suggested that mutations at four-fold degenerate sites should be completely neutral, being positions under no or limited selection. Therefore, in absence of selective constraints, the heavy (antisense) strand would accumulate G and T at these sites, while the light (sense) strand would accumulate A and C. Consequently, A-T and G-C skews at the four-fold degenerate codon sites are known to be significantly correlated with the single-strand duration during duplication, and therefore with the position of each PCG with respect to the OR of that strand [22,102]. Precise linear patterns of the percentages of each of the 4 nucleotides were found when *cox3* was used as the starting point (Figure 4), being the correlation significant for 3 nucleotides out of 4. The slope was positive for A and C, and negative for G and T. This is very similar to the findings of [102] for mammals and [15] for vermetid gastropods. These data finally point to three conclusions: (i) the strand we call “ + ” (*i.e.*, the one encoding *cox1*) is the heavy (antisense) strand, while the strand we call “-” is the light (sense) strand; (ii) the CR of *S. velum* mtDNA is located immediately before *cox3* in the UR7 region; (iii) as UR7 is located at the H/L switch, we suggest this to be the OR of both strands, working in either direction.

The region of the putative OR of the H strand encompasses UR7 and a cassette of tRNAs on the “-” strand, namely *trnM*, *trnC*, *trnY*, *trnW*, *trnQ*, and *trnE*, a situation already signaled in the family Vermetidae [15] and in the unionid *Inversidens japanensis*[22]. As shown by [109], tRNAs on one strand can sometimes work as OR in the opposite one by forming alternative secondary structures other than conventional cloverleaves. Further comparison with other bivalves is tricky because all genes are on the same strand within Amarsipobranchia, but, similarly, the CR of *Mytilus* spp. is located after a group of 7 tRNAs and the *rrnL* gene [21]; the major non-coding region (MNR) of *Mimachamys nobilis* is located after a cassette of 8 tRNAs [96]; the CR of *Meretrix* spp. is located after 7 consecutive tRNAs [39,40,110,111].

### The supranumerary ORFs within UR8

If UR7 is the putative CR on *Solemya* mtDNA, the function of UR8 remains unknown. Its length and secondary structure may have some kind of signaling function, but it is quite noteworthy that two ORFs were found here: ORF117 and ORF195. Are they functional or not? Remarkably, they span over the almost complete UR8, leaving only small unassigned nucleotide stretches of 37, 11, and 12 bp, similar to other intergenic spacers in *S. velum* mtDNA (see Additional file 9).

ORF117 has a Glimmer “raw” score comparable to other PCGs of *S. velum* mtDNA (*i.e.*, *nad4L* and *nad6*). Although it is nested in the A-T rich UR8 (70.43%), it has a lower A-T content (59.83%), so that its composition is actually different from the rest of the UR8. Moreover, notwithstanding the low (for *S. velum*) A-T content, the most used codon is UUU (Phe), which is also among the most used ones in all PCGs of this mtDNA, and therefore its codon composition is similar to that of other PCGs. The nucleotide pattern at four-fold degenerate sites is also consistent with other PCGs (Figure 4). Finally, ORF117 is almost completely located in a region of UR8 with few secondary structures (Figure 5). The presence of an homolog of ORF117 in the putative CR of *Haliotis rubra*, a species that, as above mentioned, seems to retain most ancestral features of molluscan mtDNA [33], may be related to a common origin of this ORF.

Conversely, ORF195 is not found by Glimmer and has a higher A-T content than ORF117 (73.33%); consistently, the most used codon is AAA (Lys). However, possible homology of ORF195 with membrane proteins is confirmed to some extent by the finding of a large transmembrane domain; the presence of a signal peptide constitutes a further *in silico* evidence favoring the functionality of this ORF.

It is not easy to assign to a protein a functional role only relying on bioinformatics data: expectedly, given the low homology scores and the short length of both ORFs, many different kinds of proteins and ligands were suggested by tools hosted on the @TOME 2.0 server. The presence of supranumerary ORFs in mitochondrial genomes has been reported elsewhere (f.i., [11,22,112,113]; and references therein) and they mostly are of obscure function, but they generally share either a DNA-binding motif or a transmembrane region.

The commonest hit of ORF117 was with DNA-binding domains of other polypeptides, and many of them were top-ranked using the alignments scores as a sorting criterion. The putative transmembrane region of ORF195 is 19 amminoacids long and it is found in the N-terminal part of the peptide; it is followed by 12 positively charged amminoacids (either K or R) out of 32 in the C-terminal half of the protein. Interestingly, this architecture is the same described by [22] for supranumerary sex-linked ORFs in unionid mitochondrial genomes. Breton and colleagues suggest a possible role for these ORFs, which must be involved in the complex machinery of the DUI mechanism. Present findings may confirm their claim that natural selection is working on maintaining the structure, rather than the sequence, of transmembrane supranumerary mitochondrial ORFs [22].

The presence of a putative transmembrane signaling peptide in ORF195 and the DNA-binding signal in ORF117 may suggest a regulatory role for both these proteins; moreover, their presence in the *S. velum* mtDNA might constitute an evidence of the ancestral presence of such supranumerary ORFs in all bivalves. However, it has to be noted that this remains an *in silico* analysis and that some features of ORF195 could be randomly due to the high A-T content (f.i., the signal peptide): therefore, detailed analyses of mRNA gene expression are required to shed light on these issues.

### Phylogenetic analysis and the usefulness of mitochondrial markers

The position of *Solemya* is unexpected, being nested within Gastropoda in our tree. However, node support is evidently very low (44.5), so our analysis does not point to a diphyly of Bivalvia, but rather to a polytomy at the base of the tree, including *Solemya*, *Katharina* and *Haliotis*. We may suggest that the lineage leading to Opponobranchia arose so early in the molluscan radiation that little or no phylogenetic signal of this event may be retrievable in *Solemya* mtDNA. The structural similarities of *Solemya* mtDNA to *Haliotis* and *Katharina*, and the huge differences to other bivalvian mitochondrial genomes, may have affected the clustering, hence the incorrect weak relationship to Gastropoda, which has rather to be considered an artifact.

The diphyly of Bivalvia is not a new find however (see, f.i., [114-116]), even if most recent phylogenomic studies could strongly retrieve bivalves as monophyletic [29-31]. On this aspect, [117] misinterpreted our previous results [49,51], as this is the first time we obtain bivalves as diphyletic with mitochondrial DNA; this is simply because in previous works, focusing on internal relationships of the Class, we invariably forced bivalves to be monophyletic [49,51]. It is remarkable that consistency with bivalves’ lower-level taxonomy was always maintained by our previous mtDNA analyses, a consistency which is actually lacking in [117]. On the other hand, mtDNA fails to retrieve strong phylogenetic signal for the most basal molluscan phylogenetic events, thus retrieving controversial results. Other molecular markers are needed on the issue.

Mitochondrial gene arrangement may better help in tracing basal phylogenetic relationships [23-26]. Remarkably, *Solemya* gene order connects to the *Katarina* one by a single gene inversion event (Figure 7), once again pointing out that *Solemya* belongs to a group of mollusks maintaining archaic mtDNA features. The same inversion can be traced in *N. nucleus*: the presence of the inversion can be extrapolated by the sequenced regions and therefore can be considered very likely. This may be taken as evidence for a sister group relationship between Nuculida and Solemyida, thus supporting again the monophyly of Opponobranchia. Given the questioned status of Nuculanoidea (f.i., [30,49,51,117,118]; and reference therein), it would be very interesting to obtain the complete mitochondrial genome of a species belonging to this superfamily and to compare it with the one of *S. velum*. On the other hand, gene orders of autobranchiate bivalves known so far are so highly derived and hardly connectible (if not at all) to this archaic condition that the gene order of *S. velum* is useless in tracing phylogenetic relationships between Opponobranchia and other bivalves. Only the invention of a slow-evolving autobranch bivalve mtDNA (if it exists) would help to trace Bivalvia deep phylogenetic relationships based on mtDNA gene arrangements.

## Conclusions

In previous paragraphs we extensively discussed many features of the mitochondrial genome of *S. velum*, in terms of gene/nucleotide content, strand identification, putative control region, and gene arrangement. All evidences gathered from different (and partially independent) sources point towards the same conclusion: *S. velum* retains most of the ancestral mtDNA features of conchiferans, like *H. rubra* does within gastropods. The large similarities found with *K. tunicata*, an outgroup of conchiferans, on one side, and the great differences found with other known bivalves, on the other side, lead us to polarize genomic characters and conclude that the mtDNA of *S. velum* has been probably “freezed” in a condition very similar to that of the most recent common ancestor of Opponobranchia and Autobranchia.

If this is true, the wide discontinuity between *S. velum* and Autobranchia is intriguing: in facts, no mtDNA representing an intermediate state is known to date, the only exceptions being some genomic features of Palaeoheterodonta (f.i., nucleotide skews and the proportion of unassigned nucleotides; unpublished observation). In the branch leading to Autobranchia, mitochondrial genome evolved on its own, like for the translocation of most – if not all – genes on a single strand; the decrease in A-T content; a possible tendency towards heavy changes in *atp8* gene; multiple and lineage-specific events of gene rearrangement. The evolution of Autobranchia seems to be tightly coupled with a dramatic increase of gene rearrangement events. Which factors triggered this boost of genomic evolution, while the main cladogenetic event leading to the Opponobranchia-Autobranchia split was taking place in the lower Cambrian [51]? An exhaustive answer is probably beyond the scope of this paper, but it is tempting to investigate whether the appearance of DUI played a main role in this burst [16,17]: further research on DUI evolution and the characterization of mitochondrial inheritance in Opponobranchia can surely shed more light on this issue. The recent discovery of DUI in the nuculanid *Ledella ultima*[118] is very interesting in this regard and has still to be evaluated in the light of the controversial phylogenetic position of the group.

## Competing interests

The authors declare that they have no competing interests.

## Authors’ contributions

FP participated in the design of the study and in molecular lab work, analyzed the data and drafted the manuscript; AR carried out the molecular lab work and analyzed the data; MP conceived the study and helped to draft the manuscript. All authors read and approved the final manuscript.

## Supplementary Material

Additional file 1 **Primers used in this study for Long-PCR reactions.** Primers that were used for the same experiment as a forward/reverse couple were marked with the same letter. The position of the primer annealing site on the complete molecule is reported in the “Target” column [119-121]. Click here for file

Additional file 2 **Primers used in this study for routine PCR reactions.** Primers that were used for the same experiment as a forward/reverse couple were marked with the same letter. The position of the primer annealing site on the complete molecule is reported in the “Target” column [120-123]. Click here for file

Additional file 3 The dataset used for this study. Click here for file

Additional file 4 Secondary structures of tRNAs. Click here for file

Additional file 5 **Nucleotide composition of *Solemya velum *mitochondrial genome.** Click here for file

Additional file 6 **Mitogenomic features of taxa used in this study.** %UNs, percentage of unassigned nucleotides over the total length of the genome; H, number of genes on the putative H strand; L, number of genes on the putative L strand; aa, number of amminoacids encoded by the totality of protein coding genes (excluding stop codons). The *rrnS* gene is duplicated in *C. gigas*; length of either copy is reported. Click here for file

Additional file 7 Secondary structures of regions between two consecutive PCGs. Click here for file

Additional file 8 **Codon usage in *Solemya velum *mitochondrial genome.** The total frequency of each amminoacid is reported under the three-letter/one-letter name; underlined codons correspond to anticodons of mitochondrial tRNAs. All truncated (TA-/T--) stop codons were attributed to TAA. RSCU, Relative Synonymous Codon Usage. Click here for file

Additional file 9 Unassigned regions longer than 10 bp. Click here for file

Additional file 10 **Phylogenetic Representativeness.** Test is reported as in [49] for (**a**) the original master list of mollusks taken from [93] and (**b**) a set of 100 shuffled master lists simulating taxonomical revisions. In both cases, AvTD is plotted on the left axis in the upper part of the chart and VarTD on the right one in the lower part. Sample size is plotted on *x*-axis; the greatest AvTD value (upper thick continue line), the AvTD mean (thin continue line), the AvTD 95% lower confidence limit (lower thick continue line), the VarTD 95% upper confidence limit (upper thick dashed line), the VarTD mean (thin dashed line), and the lowest VarTD value (lower thick dashed line) are shown. All these lines (with the exception of greatest AvTD and lowest VarTD) are shown as two-tailed 95% confidence limits for shuffling test (b). Sample used for this work is shown as a black diamond (AvTD)/circle (VarTD). Click here for file

Additional file 11 Amminoacid substitution models selected by ProtTest 3.2 [80] and piped to RAxML 7.2.8 [82,83]. Click here for file

## Aknowledgements

We would like to thank Jeffrey L. Boore for providing specimens of *S. velum* used for this study and for the stimulating discussions on mitochondrial genomics with MP. This work was supported by the University and Research Italian Ministry (MIUR PRIN07, grant number 2007NSHJL8_002, and PRIN09, grant number 2009NWXMXX_002) and the “Canziani Bequest” fund (University of Bologna, grant number A.31.CANZELSEW). FP was financially supported by Fondazione del Monte di Bologna e Ravenna.
