# Supplementary material for: The complete mitochondrial genome of Solemya velum (Mollusca: Bivalvia) and its relationships with Conchifera
Source: BMC Genomics. 2013 Jun 18;14:409. doi: 10.1186/1471-2164-14-409 (PMC3704766; doi:10.1186/1471-2164-14-409)
Supplement: Additional file 1 — Primers used in this study for Long-PCR reactions. Primers that were used for the same experiment as a forward/reverse couple were marked with the same letter. The position of the primer annealing site on the complete molecule is reported in the “Target” column [119-121]. [file 1471-2164-14-409-S1.doc]

| Primer name | Sequence 5'-3' | Lenght (bp) | Target | Annealing | Reference |
| --- | --- | --- | --- | --- | --- |
| SR-N-14745 ^a^ | GTGCCAGCAGYYGCGGTTANAC | 22 | 11801-11780 | 48°C | [119] |
| 16SbrH(32) ^a^ | CCGGTCTGAACTCAGATCACGT | 22 | 9845-9866 | 48°C | [120] |
| COI2F ^b^ | TGAGCCGGTATAGTTGGAACATC | 23 | 64-86 | 48°C | [121] |
| Solemya_3a1894R ^b^ | GAGCGGAAAGGTTTAGGTTATT | 22 | 9495-9474 | 48°C | This study |
| Sol-16S317R ^c^ | CAAGAAGACCCTATTGAGCTTTAGT | 25 | 10172-10148 | 52°C | This study |
| Sol-cob239R ^c^ | AACTACAATAAAGCCAACCAAGTCC | 25 | 7342-7366 | 52°C | This study |
| Sol-12S213F ^d^ | CACCTACTTTGTTACGACTTATCT | 24 | 11180-11203 | 53.5°C | This study |
| Sol-cox14673R ^d^ | AATCATCTGAATACTTTAATTCCA | 24 | 962-939 | 53.5°C | This study |
| SoVe-12S-59F ^e^ | CTTACACCATATTACAGGGACTAC | 24 | 12379-12402 | 48°C | This study |
