# Supplementary material for: The complete mitochondrial genome of Solemya velum (Mollusca: Bivalvia) and its relationships with Conchifera
Source: BMC Genomics. 2013 Jun 18;14:409. doi: 10.1186/1471-2164-14-409 (PMC3704766; doi:10.1186/1471-2164-14-409)
Supplement: Additional file 2 — Primers used in this study for routine PCR reactions. Primers that were used for the same experiment as a forward/reverse couple were marked with the same letter. The position of the primer annealing site on the complete molecule is reported in the “Target” column [120-123]. [file 1471-2164-14-409-S2.doc]

| Primer name | Sequence 5'-3' | Lenght (bp) | Target | Annealing | Reference |
| --- | --- | --- | --- | --- | --- |
| COIF ^a^ | ATYGGNGGNTTYGGNAAYTG | 20 | 214-233 | 48°C | [122] |
| COIR ^a^ | ATNGCRAANACNGCNCCYAT | 20 | 1157-1138 | 48°C | [122] |
| cox3F ^b, h^ | TGGTGGCGAGATGTKKTNCGNGA | 23 | 12798-12820 | 48°C b/53°C ^h^ | [123] |
| cox3R ^b, g^ | ACWACGTCKACGAAGTGTCARTATCA | 26 | 13372-13347 | 48°C b/53°C ^g^ | [123] |
| 16SbrH(32) ^c^ | CCGGTCTGAACTCAGATCACGT | 22 | 9845-9866 | 48°C | [120] |
| 16Sar(34) ^c^ | CGCCTGTTTAACAAAAACAT | 20 | 10399-10380 | 48°C | [120] |
| COI2F ^d^ | TGAGCCGGTATAGTTGGAACATC | 23 | 64-86 | 50°C | [121] |
| COI546R ^d, h^ | ATTGCTCCGGCTAGAACTGGAAGT | 24 | 608-585 | 50°C d/53°C ^h^ | [121] |
| Solemya_3a621F ^e^ | TTCAAAACCGGATGATTTTTAC | 22 | 8012-8033 | 52°C | This study |
| Solemya_3a1894R ^e^ | GAGCGGAAAGGTTTAGGTTATT | 22 | 9495-9474 | 52°C | This study |
| Sol-c444F ^f^ | GCAGGCTCTACTTTTCATTT | 20 | 10343-10362 | 50°C | This study |
| Sol-c1349R ^f^ | TGTGTGATTCGATTTATTTTG | 21 | 11109-11089 | 50°C | This study |
| Sol-12S213F ^g^ | CACCTACTTTGTTACGACTTATCT | 24 | 11180-11203 | 53°C | This study |
| SoVe-cox1-2559R ^i^ | CTTTTCTCCTTTATTCAGAGGTA | 23 | 15285-15263 | 54°C | This study |
| SoVe-cox3-663F ^i, m, n^ | TAAAATACAACAAAAATACACCAA | 24 | 13509-13532 | 54°C ^i, m, n^ | This study |
| SoVe-C12587F ^j^ | GATATTAGCCAAAACCAAACTAA | 23 | 12019-12041 | 54°C | This study |
| SoVe-C12587R ^j^ | TGAGTTATAGGCGATAGATTGTAG | 24 | 12241-12218 | 54°C | This study |
| SoVe-TTT13785F ^k^ | TTCTTGTAGGAACTTCATTTCTATT | 25 | 13252-13276 | 54°C | This study |
| SoVe-TTT13785R ^k^ | GATTAAAAATCTAACGCTTATTCTC | 25 | 13454-13430 | 54°C | This study |
| SoVe-SEQ31-F ^l^ | ATTAAAATTGGCCTAACTCCT | 21 | 14924-14944 | 54°C | This study |
| SoVe-SEQ32-R ^l^ | AAAGTATCTTGAAGTTGATAGTGG | 24 | 15583-15560 | 54°C | This study |
| SoVe-cox1bis-1199R ^m^ | AATTATAGGGGATATAAACATTCA | 24 | 14591-14568 | 54°C | This study |
