# Supplementary material for: The complete mitochondrial genome of Solemya velum (Mollusca: Bivalvia) and its relationships with Conchifera
Source: BMC Genomics. 2013 Jun 18;14:409. doi: 10.1186/1471-2164-14-409 (PMC3704766; doi:10.1186/1471-2164-14-409)
Supplement: Additional file 3 — The dataset used for this study. [file 1471-2164-14-409-S3.doc]

| Species | Class | Subclass | Order | Family | GenBank Accession Number |
| --- | --- | --- | --- | --- | --- |
| *Acanthocardia tuberculata* | Bivalvia | Heterodonta | Chamida | Cardiidae | [GenBank:DQ632743] |
| *Sinonovacula constricta* | Bivalvia | Heterodonta | Chamida | Psammobiidae | [GenBank:EU880278] |
| *Paphia euglypta* | Bivalvia | Heterodonta | Chamida | Veneridae | [GenBank:GU269271] |
| *Venerupis philippinarum* F | Bivalvia | Heterodonta | Chamida | Veneridae | [GenBank:NC_003354] |
| *Hiatella arctica* | Bivalvia | Heterodonta | Hiatelloidea | Hiatellidae | [GenBank:DQ632742] |
| *Loripes lacteus* | Bivalvia | Heterodonta | Veneroida | Lucinidae | [GenBank:EF043341] |
| *Lucinella divaricata* | Bivalvia | Heterodonta | Veneroida | Lucinidae | [GenBank:EF043342] |
| *Meretrix petechialis* | Bivalvia | Heterodonta | Veneroida | Veneridae | [GenBank:NC_012767] |
| *Margaritifera falcata* | Bivalvia | Palaoheterodonta | Unionida | Margaritiferidae | [GenBank:NC_015476] |
| *Cristaria plicata* | Bivalvia | Palaoheterodonta | Unionida | Unionidae | [GenBank:FJ986302] |
| *Hyriopsis schlegelii* F | Bivalvia | Palaoheterodonta | Unionida | Unionidae | [GenBank:HQ641406] |
| *Lampsilis ornata* | Bivalvia | Palaoheterodonta | Unionida | Unionidae | [GenBank:AY365193] |
| *Lasmigona compressa* | Bivalvia | Palaoheterodonta | Unionida | Unionidae | [GenBank:NC_015481] |
| *Pyganodon grandis* F | Bivalvia | Palaoheterodonta | Unionida | Unionidae | [GenBank:FJ809754] |
| *Quadrula quadrula* F | Bivalvia | Palaoheterodonta | Unionida | Unionidae | [GenBank:FJ809750] |
| *Sinanodonta woodiana* | Bivalvia | Palaoheterodonta | Unionida | Unionidae | [GenBank:HQ283345] |
| *Unio pictorum* | Bivalvia | Palaoheterodonta | Unionida | Unionidae | [GenBank:NC_015310] |
| *Utterbackia peninsularis* | Bivalvia | Palaoheterodonta | Unionida | Unionidae | [GenBank:NC_015477] |
| *Venustaconcha ellipsiformis* F | Bivalvia | Palaoheterodonta | Unionida | Unionidae | [GenBank:FJ809753] |
| *Solemya velum* | Bivalvia | Protobranchia | Solemyoida | Solemyidae | [GenBank:NC_017612] |
| *Musculista senhousia* F | Bivalvia | Pteriomorphia | Mytilida | Mytilidae | [GenBank:GU001953] |
| *Mytilus galloprovincialis* F | Bivalvia | Pteriomorphia | Mytilida | Mytilidae | [GenBank:AY497292] |
| *Crassostrea gigas* | Bivalvia | Pteriomorphia | Ostreoida | Ostreidae | [GenBank:NC_001276] |
| *Ostrea edulis* | Bivalvia | Pteriomorphia | Ostreoida | Ostreidae | [GenBank:JF274008] |
| *Saccostrea mordax* | Bivalvia | Pteriomorphia | Ostreoida | Ostreidae | [GenBank:FJ841968] |
| *Argopecten irradians* | Bivalvia | Pteriomorphia | Ostreoida | Pectinidae | [GenBank:EU023915] |
| *Chlamys farreri* | Bivalvia | Pteriomorphia | Ostreoida | Pectinidae | [GenBank:EF473269] |
| *Mimachlamys nobilis* | Bivalvia | Pteriomorphia | Ostreoida | Pectinidae | [GenBank:FJ415225] |
| *Mizuhopecten yessoensis* | Bivalvia | Pteriomorphia | Ostreoida | Pectinidae | [GenBank:FJ595959] |
| *Placopecten magellanicus* | Bivalvia | Pteriomorphia | Ostreoida | Pectinidae | [GenBank:DQ088274] |
| *Chaetoderma nitidulum* | Caudofoveata |  | Chaetodermatida | Chaetodermatidae | [GenBank:EF211990] |
| *Sepia officinalis* | Cephalopoda | Coleoida | Sepiida | Sepiidae | [GenBank:AB240155] |
| *Architeuthis dux* | Cephalopoda | Coleoida | Teuthida | Architeuthidae | [GenBank:NC_011581] |
| *Loligo bleekeri* | Cephalopoda | Coleoida | Teuthida | Loliginidae | [GenBank:NC_002507] |
| *Dosidicus gigas* | Cephalopoda | Coleoida | Teuthida | Ommastrephidae | [GenBank:NC_009734] |
| *Sthenoteuthis oualaniensis* | Cephalopoda | Coleoida | Teuthida | Ommastrephidae | [GenBank:NC_010636] |
| *Nautilus macromphalus* | Cephalopoda | Nautiloida | Nautilida | Nautilidae | [GenBank:NC_007980] |
| *Biomphalaria glabrata* | Gastropoda | Divasibranchia | Basommatophora | Planorbidae | [GenBank:NC_005439] |
| *Siphonaria pectinata* | Gastropoda | Divasibranchia | Basommatophora | Siphonariidae | [GenBank:NC_012383] |
| *Cepaea nemoralis* | Gastropoda | Divasibranchia | Geophila | Helicidae | [GenBank:NC_001816] |
| *Haliotis rubra* | Gastropoda | Euomphalomorpha | Vetigastropoda | Haliotidae | [GenBank:NC_005940] |
| *Onchidella celtica* | Gastropoda | Gymnomorpha | Onchidiida | Onchidiidae | [GenBank:NC_012376] |
| *Pyramidella dolabrata* | Gastropoda | Heterobranchia | Pyramidelloida | Pyramidellidae | [GenBank:NC_012435] |
| *Aplysia californica* | Gastropoda | Opisthobranchia | Anaspidea | Aplysiidae | [GenBank:NC_005827] |
| *Pupa strigosa* | Gastropoda | Opisthobranchia | Cephalaspidea | Acteonidae | [GenBank:NC_002176] |
| *Micromelo undata* | Gastropoda | Opisthobranchia | Cephalaspidea | Hydatinidae | [GenBank:NC_015106] |
| *Berthellina* sp. | Gastropoda | Opisthobranchia | Notapsidea | Pleurobranchidae | [GenBank:NC_015091] |
| *Notodoris gardineri* | Gastropoda | Opisthobranchia | Nudibranchia | Aegiretidae | [GenBank:NC_015111] |
| *Elysia chlorotica* | Gastropoda | Opisthobranchia | Sacoglossa | Plakobranchidae | [GenBank:NC_010567] |
| *Cancellaria cancellata* | Gastropoda | Orthogastropoda | Neogastropoda | Cancellariidae | [GenBank:NC_013241] |
| *Bolinus brandaris* | Gastropoda | Orthogastropoda | Neogastropoda | Muricidae | [GenBank:NC_013250] |
| *Nassarius reticulatus* | Gastropoda | Orthogastropoda | Neogastropoda | Nassariidae | [GenBank:NC_013248] |
| *Conus textile* | Gastropoda | Orthogastropoda | Neogastropoda | Terebridae | [GenBank:NC_008797] |
| *Terebra dimidiata* | Gastropoda | Orthogastropoda | Neogastropoda | Terebridae | [GenBank:NC_013239] |
| *Fusiturris similis* | Gastropoda | Orthogastropoda | Neogastropoda | Turridae | [GenBank:NC_013242] |
| *Cymbium olla* | Gastropoda | Orthogastropoda | Neogastropoda | Volutidae | [GenBank:NC_013245] |
| *Cymatium parthenopeum* | Gastropoda | Orthogastropoda | Neomesogastropoda | Ranellidae | [GenBank:NC_013247] |
| *Oncomelania hupensis* | Gastropoda | Orthogastropoda | Neotaenioglossa | Pomatiopsidae | [GenBank:NC_013073] |
| *Dendropoma gregarium* | Gastropoda | Orthogastropoda | Neotaenioglossa | Vermetidae | [GenBank:NC_014580] |
| *Myosotella myosotis* | Gastropoda | Pulmonata | Archaeopulmonata | Ellobiidae | [GenBank:NC_012434] |
| *Katharina tunicata* | Polyplacophora |  | Lepidopleurida | Mopaliidae | [GenBank:KTU09810] |
| *Graptacme eborea* | Scaphopoda |  | Dentaliida | Dentaliidae | [GenBank:AY484748] |
| *Platynereis dumerilii* | Polychaeta | Aciculata | Phyllodocida | Nereidae | [GenBank:NC_000931] |
