# Supplementary material for: The complete mitochondrial genome of Solemya velum (Mollusca: Bivalvia) and its relationships with Conchifera
Source: BMC Genomics. 2013 Jun 18;14:409. doi: 10.1186/1471-2164-14-409 (PMC3704766; doi:10.1186/1471-2164-14-409)
Supplement: Additional file 4 — Secondary structures of tRNAs. [file 1471-2164-14-409-S4.pdf]

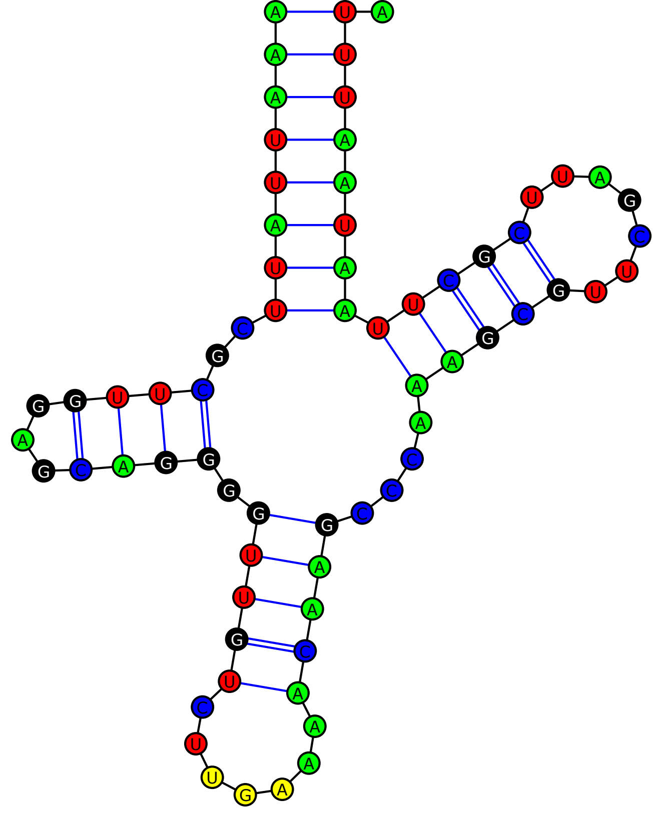
tRNA-Ser2(UGA)

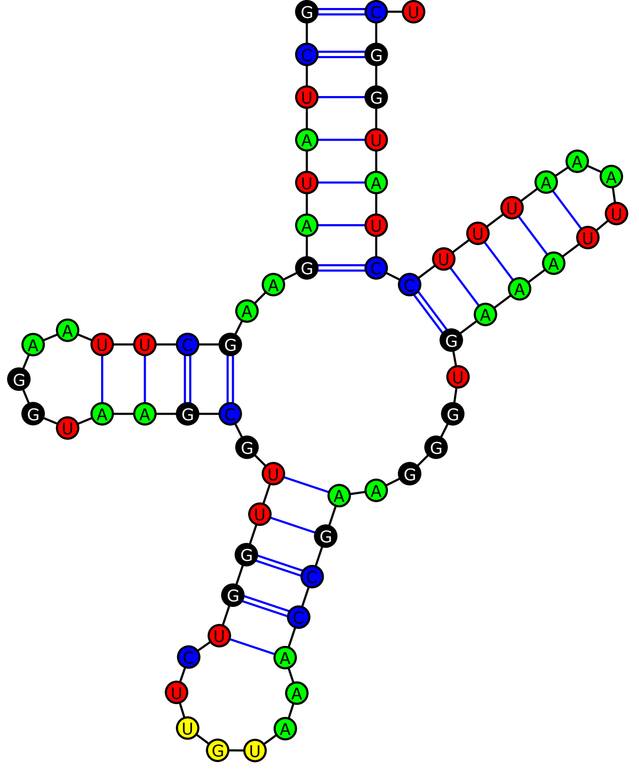
tRNA-Thr(UGU)

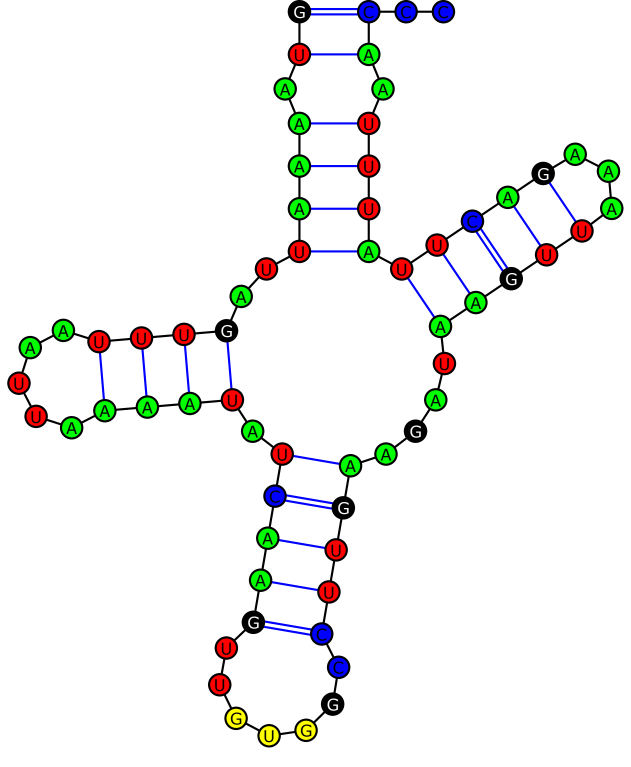
tRNA-His(GUG)

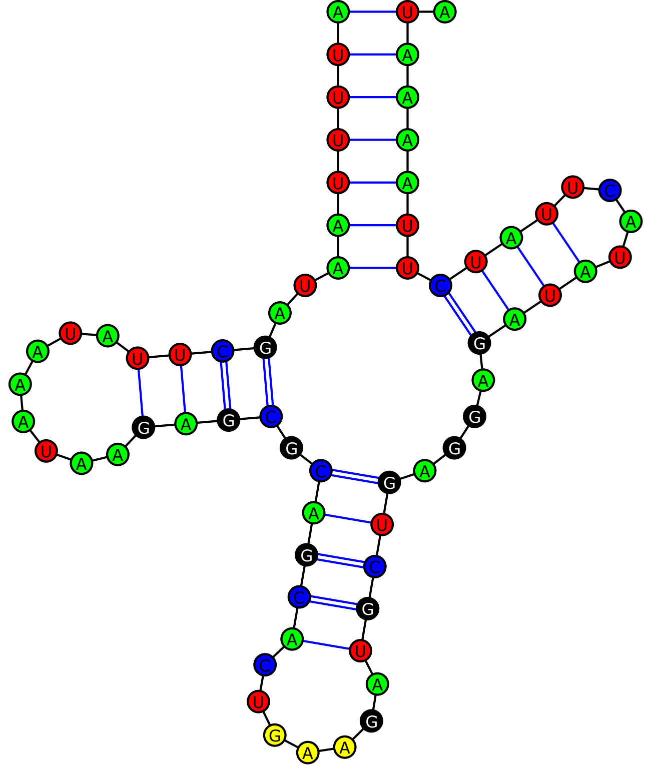
tRNA-Phe(GAA)

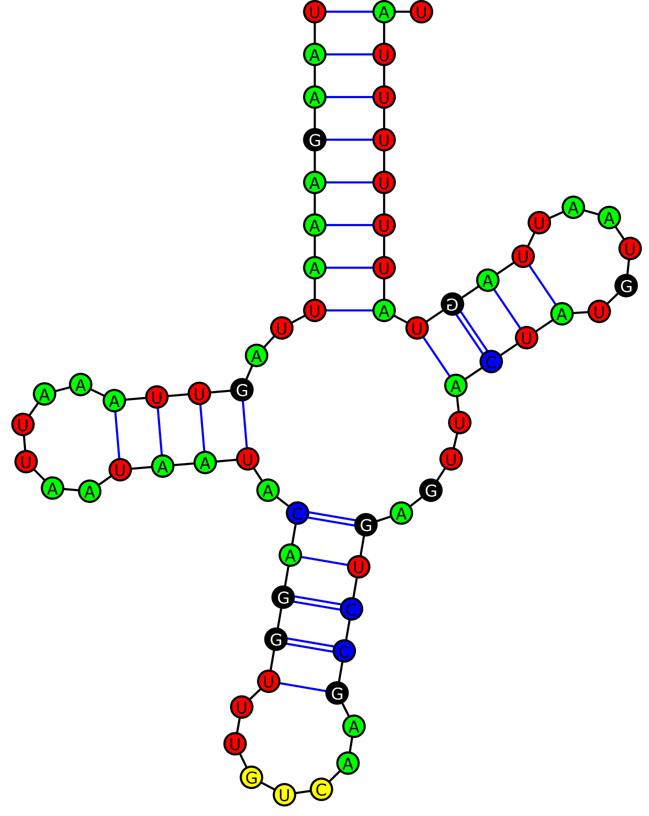
tRNA-Asp(GUC)

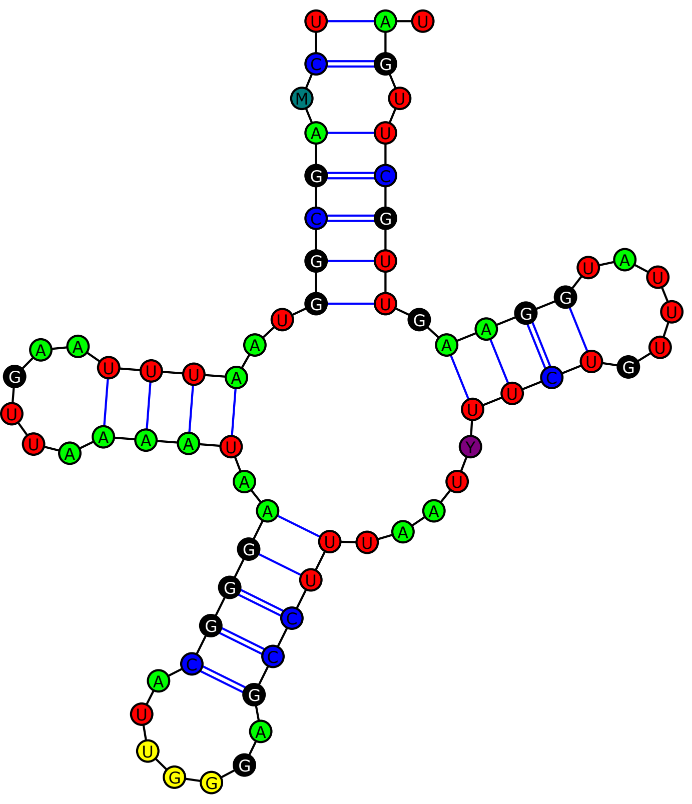
tRNA-Pro(UGG)

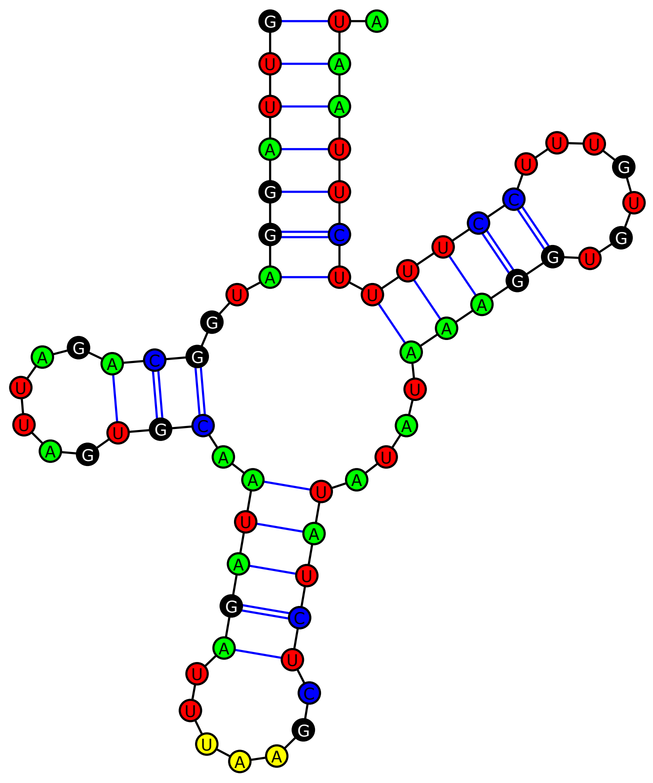
tRNA-Leu2(UAA)

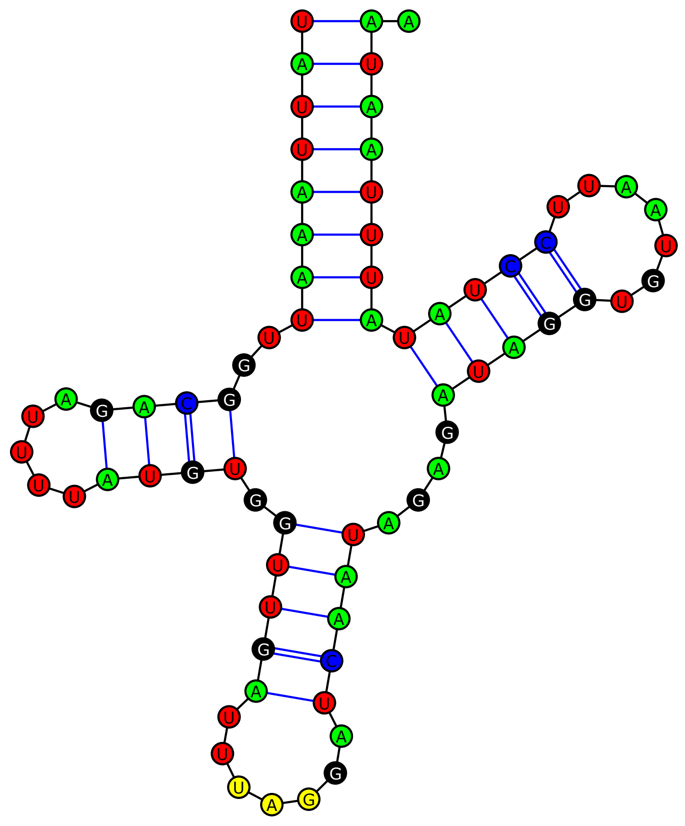
tRNA-Leu1(UAG)

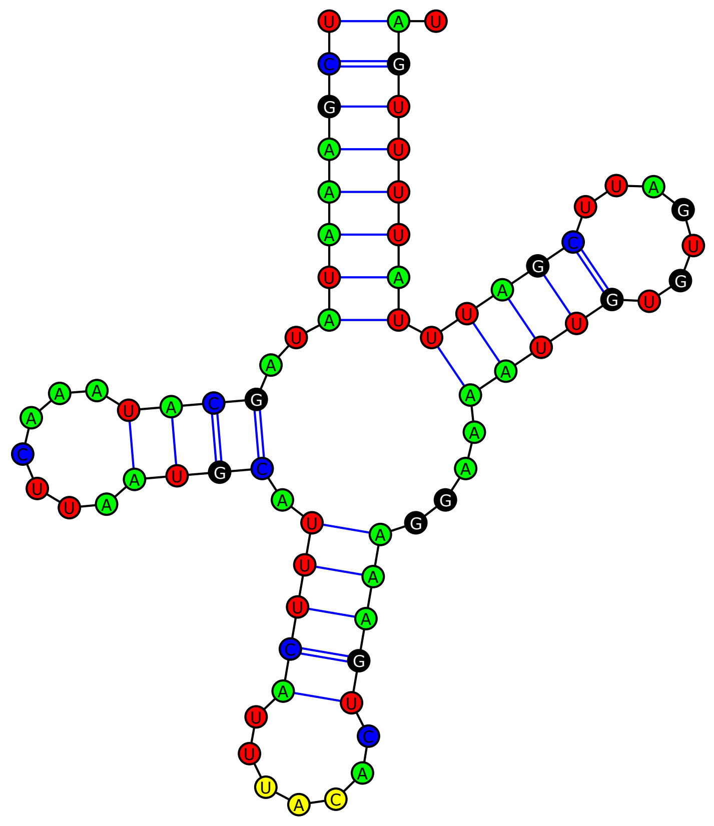
tRNA-Val(UAC)

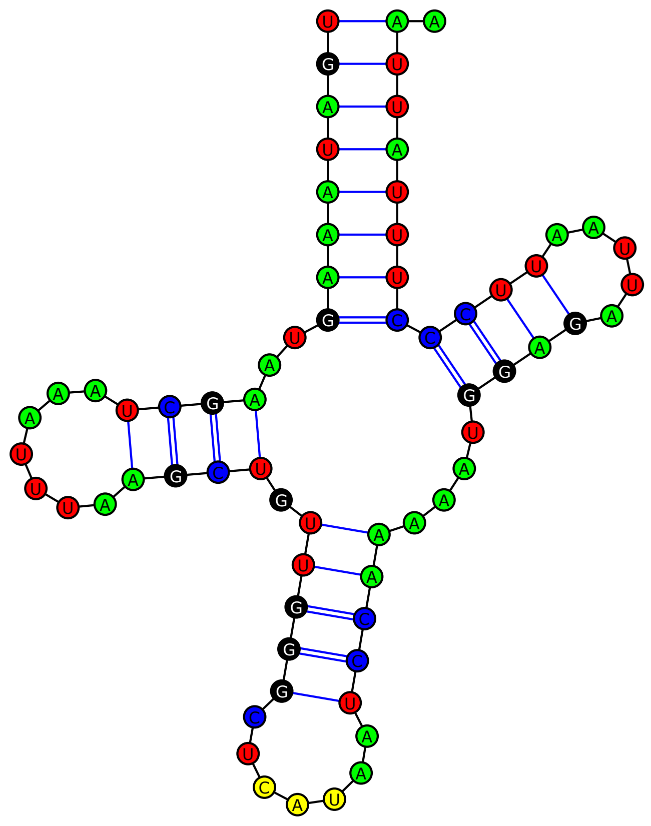
tRNA-Met(CAU)

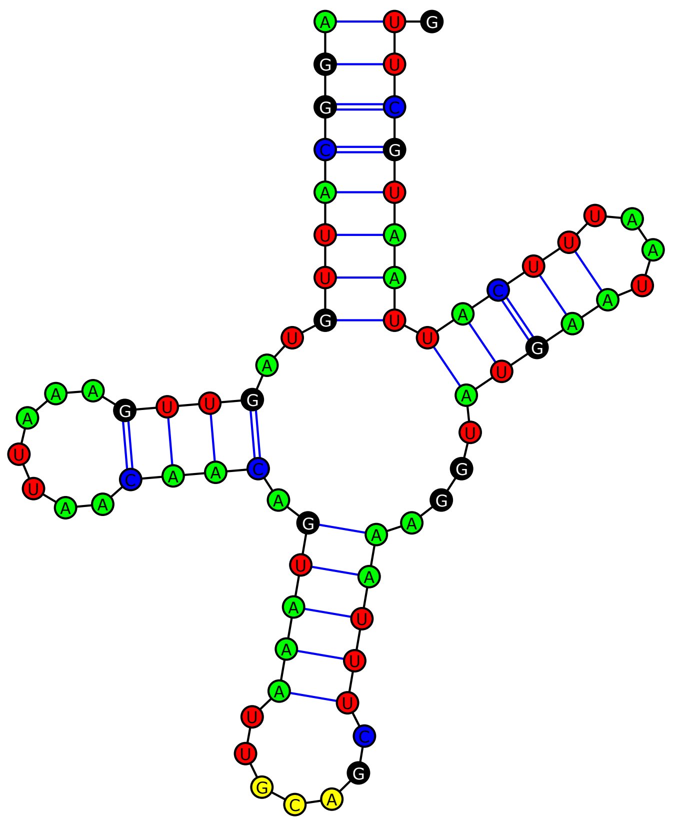
tRNA-Cys(GCA)

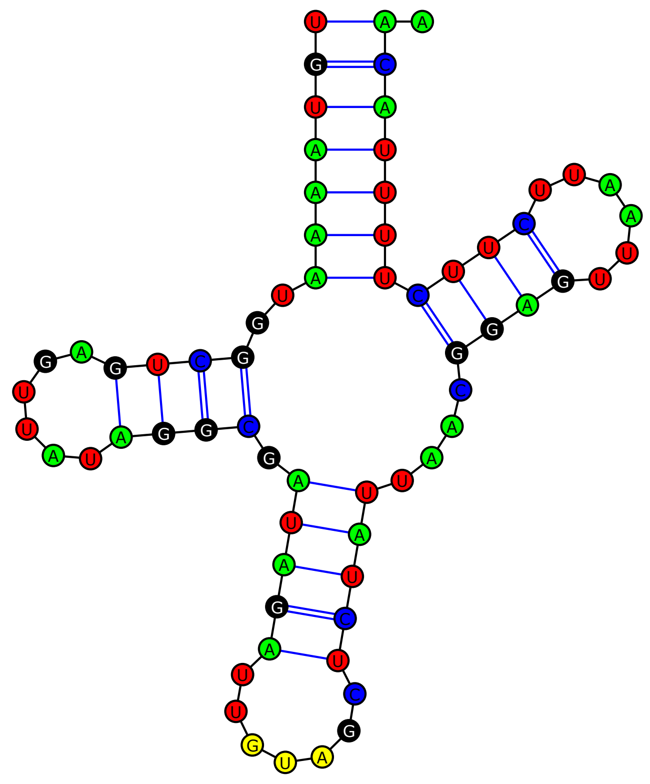
tRNA-Tyr(GUA)

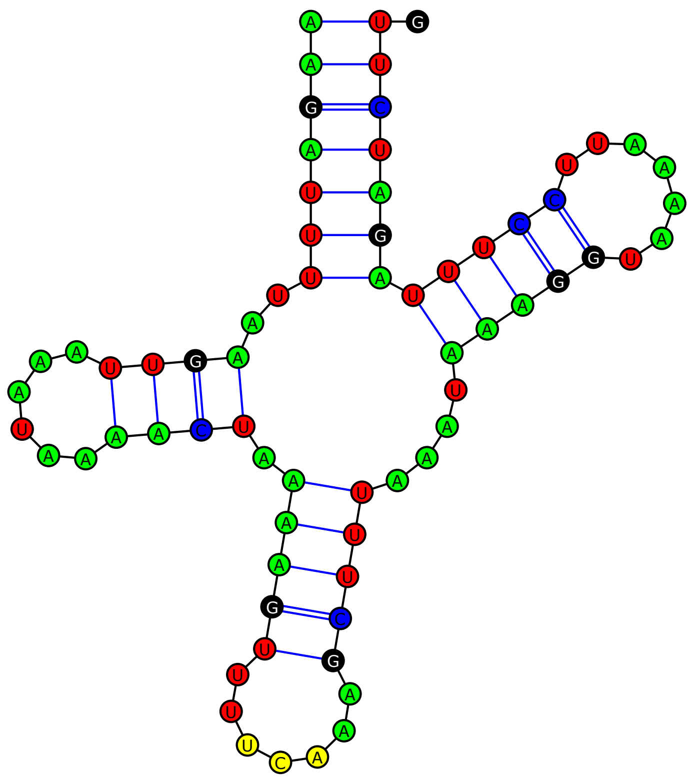
tRNA-Trp(UCA)

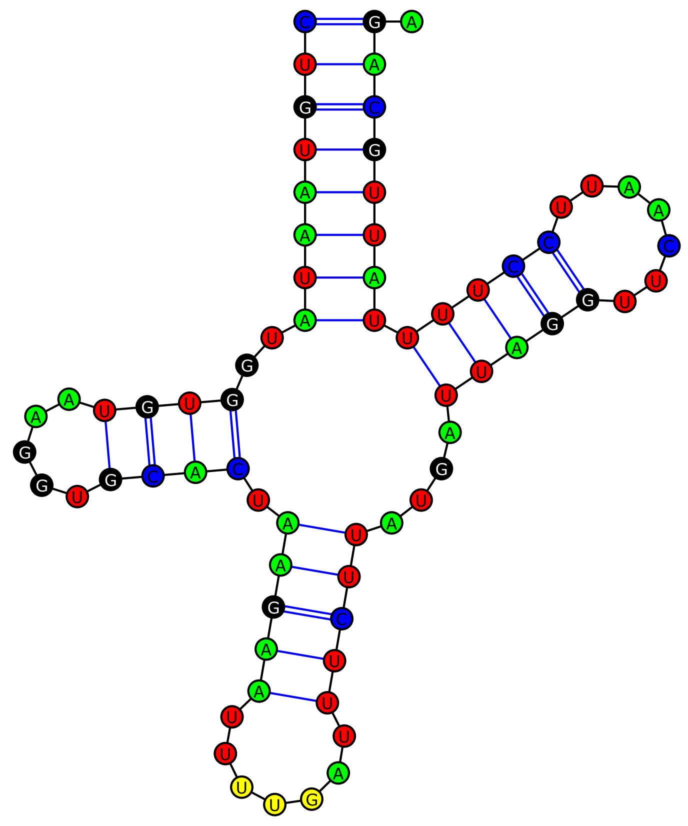
tRNA-Gln(UUG)

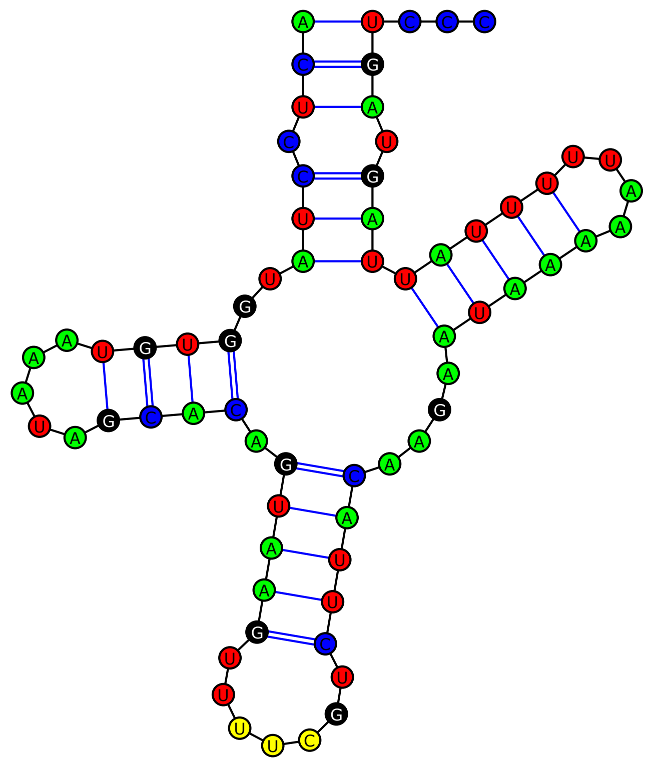
tRNA-Glu(UUC)

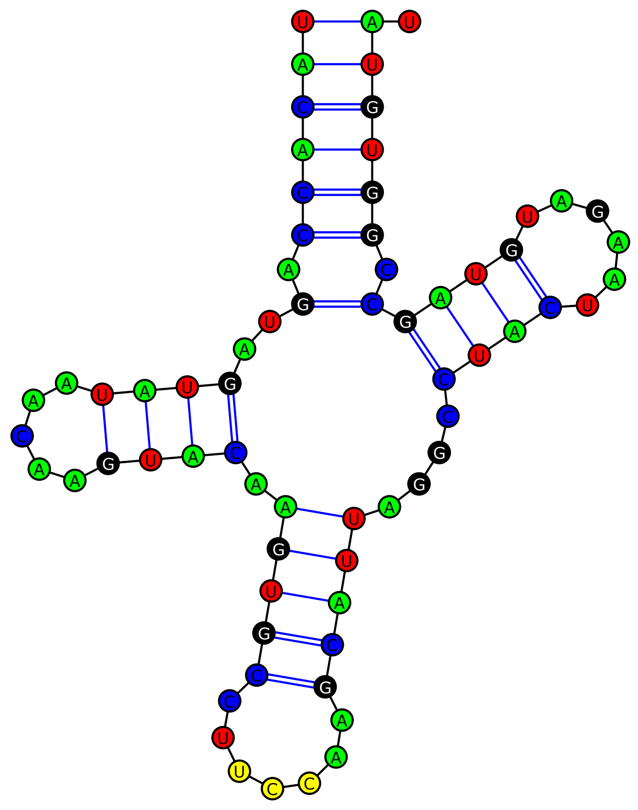
tRNA-Gly(UCC)

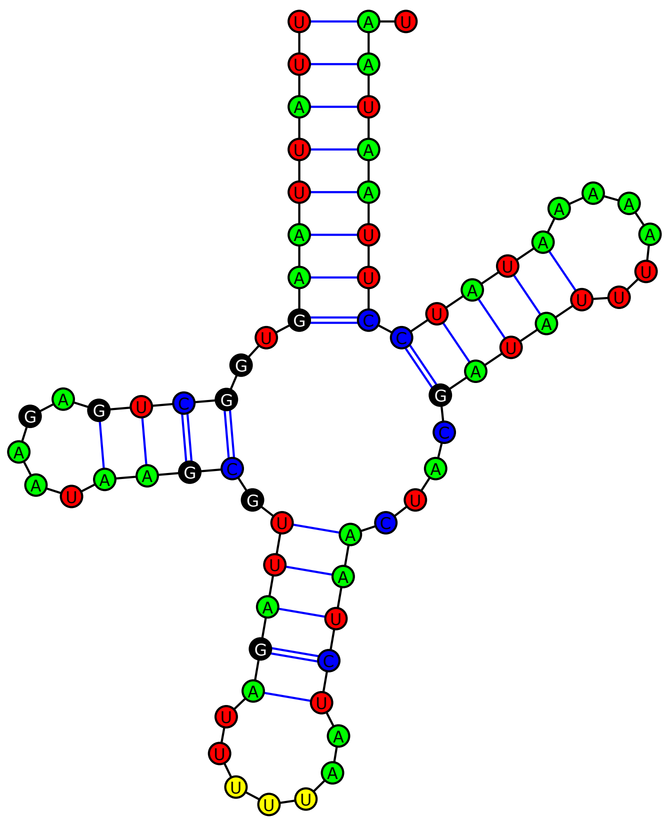
tRNA-Lys(UUU)

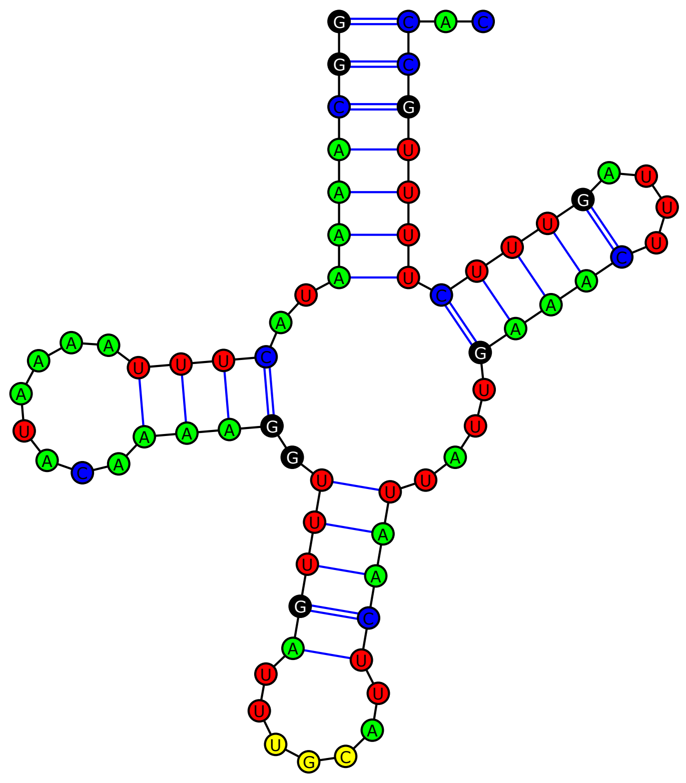
tRNA-Ala(UGC)

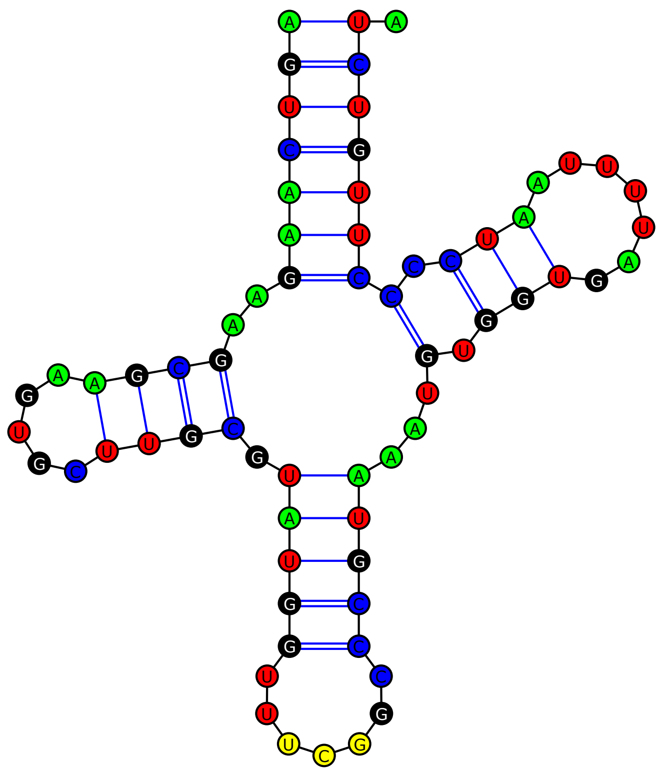
tRNA-Arg(UCG)

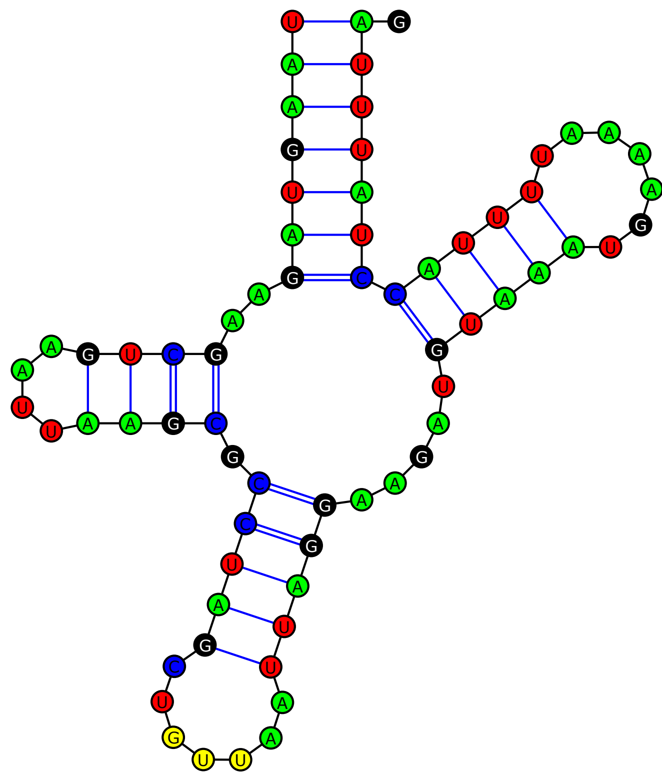
tRNA-Asn(GUU)

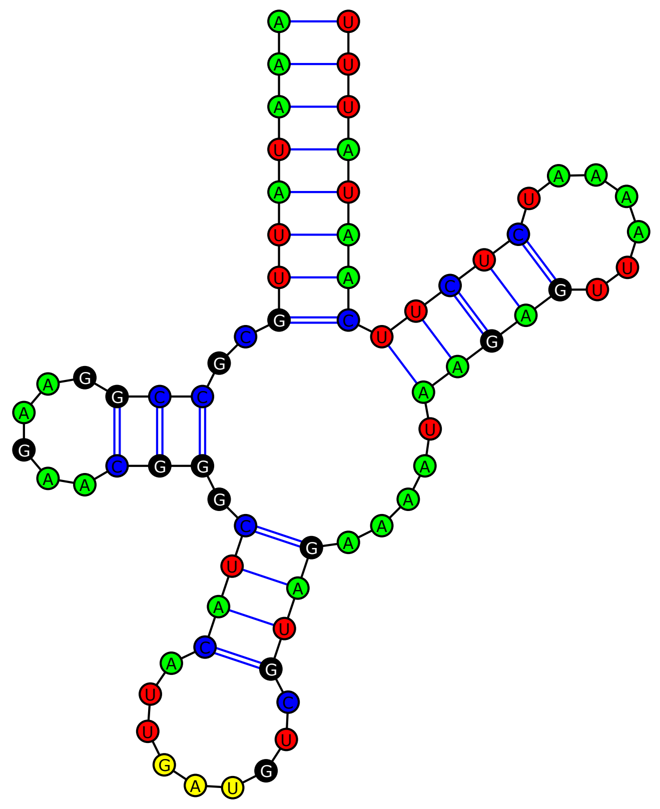
tRNA-Ile(GAU)

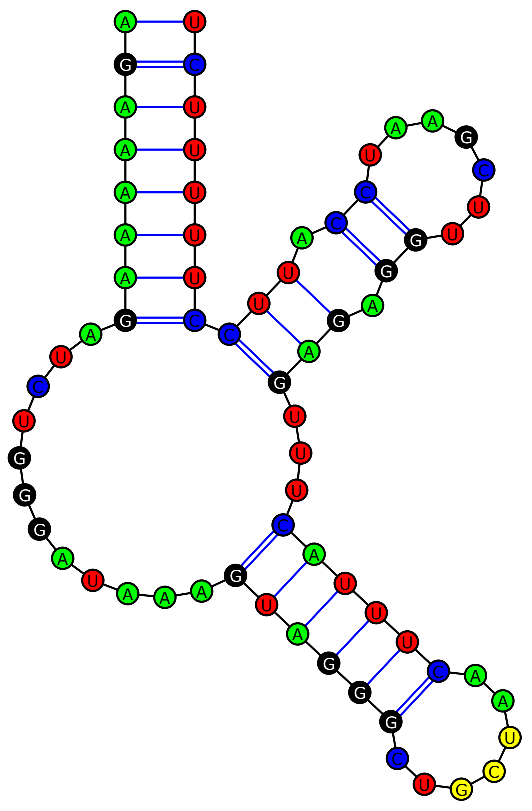
tRNA-Ser1(GCU)
