# Supplementary material for: The complete mitochondrial genome of Solemya velum (Mollusca: Bivalvia) and its relationships with Conchifera
Source: BMC Genomics. 2013 Jun 18;14:409. doi: 10.1186/1471-2164-14-409 (PMC3704766; doi:10.1186/1471-2164-14-409)
Supplement: Additional file 5 — Nucleotide composition of Solemya velum mitochondrial genome. [file 1471-2164-14-409-S5.doc]

| Gene/region | Length (bp) | %T(U) | %C | %A | %G | %A+T | %G+C |
| --- | --- | --- | --- | --- | --- | --- | --- |
| *rrnS* | 885 | 33.79 | 18.98 | 35.14 | 12.09 | 68.93 | 31.07 |
| *rrnL* | 1376 | 34.30 | 19.40 | 36.26 | 10.03 | 70.57 | 29.43 |
| *atp6* | 696 | 23.71 | 18.82 | 46.26 | 11.21 | 69.97 | 30.03 |
| *atp8* | 165 | 30.30 | 20.61 | 41.21 | 7.88 | 71.52 | 28.48 |
| *cox1* | 1536 | 33.33 | 20.12 | 29.23 | 17.32 | 62.57 | 37.43 |
| *cox2* | 687 | 34.06 | 17.90 | 30.42 | 17.61 | 64.48 | 35.52 |
| *cox3* | 780 | 33.97 | 20.51 | 26.03 | 19.49 | 60.00 | 40.00 |
| *cytb* | 1123 | 23.78 | 18.97 | 43.90 | 13.36 | 67.68 | 32.32 |
| *nad1* | 942 | 23.78 | 21.44 | 44.80 | 9.98 | 68.58 | 31.42 |
| *nad2* | 1089 | 37.83 | 16.53 | 30.85 | 14.78 | 68.69 | 31.31 |
| *nad3* | 353 | 38.24 | 17.28 | 28.90 | 15.58 | 67.14 | 32.86 |
| *nad4* | 1344 | 36.09 | 18.38 | 33.18 | 12.35 | 69.27 | 30.73 |
| *nad4L* | 297 | 39.73 | 16.50 | 31.65 | 12.12 | 71.38 | 28.62 |
| *nad5* | 1727 | 34.22 | 19.46 | 34.16 | 12.16 | 68.38 | 31.62 |
| *nad6* | 501 | 23.15 | 20.76 | 47.50 | 8.58 | 70.66 | 29.34 |
| *trnA* | 70 | 38.57 | 14.29 | 34.29 | 12.86 | 72.86 | 27.14 |
| *trnR* | 68 | 32.35 | 19.12 | 22.06 | 26.47 | 54.41 | 45.59 |
| *trnN* | 69 | 31.88 | 10.14 | 37.68 | 20.29 | 69.57 | 30.43 |
| *trnD* | 69 | 36.23 | 14.49 | 42.03 | 7.25 | 78.26 | 21.74 |
| *trnC* | 70 | 37.14 | 18.57 | 34.29 | 10.00 | 71.43 | 28.57 |
| *trnQ* | 71 | 26.76 | 19.72 | 42.25 | 11.27 | 69.01 | 30.99 |
| *trnE* | 68 | 33.82 | 14.71 | 35.29 | 16.18 | 69.12 | 30.88 |
| *trnG* | 68 | 25.00 | 22.06 | 32.35 | 20.59 | 57.35 | 42.65 |
| *trnH* | 66 | 34.85 | 10.61 | 39.39 | 15.15 | 74.24 | 25.76 |
| *trnI* | 70 | 28.57 | 14.29 | 37.14 | 20.00 | 65.71 | 34.29 |
| *trnL1* | 71 | 33.80 | 19.72 | 40.85 | 5.63 | 74.65 | 25.35 |
| *trnL2* | 68 | 29.41 | 20.59 | 39.71 | 10.29 | 69.12 | 30.88 |
| *trnK* | 70 | 38.57 | 10.00 | 38.57 | 12.86 | 77.14 | 22.86 |
| *trnM* | 69 | 37.68 | 15.94 | 33.33 | 13.04 | 71.01 | 28.99 |
| *trnF* | 67 | 29.85 | 11.94 | 40.30 | 17.91 | 70.15 | 29.85 |
| *trnP* | 69 | 27.54 | 24.64 | 37.68 | 10.14 | 65.22 | 34.78 |
| *trnS1* | 69 | 33.33 | 15.94 | 28.99 | 21.74 | 62.32 | 37.68 |
| *trnS2* | 69 | 30.43 | 17.39 | 30.43 | 21.74 | 60.87 | 39.13 |
| *trnT* | 66 | 30.30 | 25.76 | 30.30 | 13.64 | 60.61 | 39.39 |
| *trnW* | 70 | 44.29 | 11.43 | 35.71 | 8.57 | 80.00 | 20.00 |
| *trnY* | 67 | 29.85 | 20.90 | 37.31 | 11.94 | 67.16 | 32.84 |
| *trnV* | 73 | 35.62 | 15.07 | 38.36 | 10.96 | 73.97 | 26.03 |
| Genome | 15660 | 32.58 | 18.74 | 35.53 | 13.14 | 68.11 | 31.89 |
| PCGs | 11240 | 38.21 | 16.40 | 28.92 | 16.47 | 67.14 | 32.86 |
| PCGs (3rd) | 3746 | 38.32 | 12.39 | 38.93 | 10.36 | 77.25 | 22.75 |
| rRNAs | 2261 | 35.82 | 10.84 | 34.10 | 19.24 | 69.92 | 30.08 |
| tRNAs | 1517 | 34.61 | 13.25 | 34.28 | 17.86 | 68.89 | 31.11 |
| URs | 645 | 39.84 | 15.19 | 37.21 | 7.75 | 77.05 | 22.95 |
