# Supplementary material for: The complete mitochondrial genome of Solemya velum (Mollusca: Bivalvia) and its relationships with Conchifera
Source: BMC Genomics. 2013 Jun 18;14:409. doi: 10.1186/1471-2164-14-409 (PMC3704766; doi:10.1186/1471-2164-14-409)
Supplement: Additional file 6 — Mitogenomic features of taxa used in this study. %UNs, percentage of unassigned nucleotides over the total length of the genome; H, number of genes on the putative H strand; L, number of genes on the putative L strand; aa, number of amminoacids encoded by the totality of protein coding genes (excluding stop codons). The rrnS gene is duplicated in C. gigas; length of either copy is reported. [file 1471-2164-14-409-S6.doc]

| Species | Class |  | Genome features | | | | | PCGs | | *rrnS* | | *rrnL* | |
| --- | --- | --- | --- | --- | --- | --- | --- | --- | --- | --- | --- | --- | --- |
|  |  | Length  (bp) | | A+T  (%) | %UNs | H | L | aa | A+T  (%) | Length  (bp) | A+T  (%) | Length  (bp) | A+T  (%) |
| *Acanthocardia tuberculata* | Bivalvia | 16,104 | | 59.90 | 10.16 | 37 | 0 | 3636 | 59.50 | 824 | 59.47 | 1213 | 65.21 |
| *Aplysia californica* | Gastropoda | 14,117 | | 66.37 | 0.48 | 24 | 13 | 3618 | 66.71 | 725 | 65.38 | 1026 | 67.64 |
| *Architeuthis dux* | Cephalopoda | 20,331 | | 69.72 | 7.01 | 21 | 22 | 4733 | 68.39 | 916 | 73.25 | 1407 | 73.49 |
| *Argopecten irradians* | Bivalvia | 16,221 | | 56.93 | 8.54 | 36 | 0 | 3703 | 56.67 | 908 | 56.83 | 1347 | 59.54 |
| *Berthellina* sp. | Gastropoda | 15,688 | | 67.78 | 9.98 | 24 | 13 | 3593 | 67.37 | 741 | 66.53 | 1097 | 63.45 |
| *Biomphalaria glabrata* | Gastropoda | 13,670 | | 74.63 | 0.00 | 24 | 13 | 3549 | 74.20 | 708 | 74.72 | 981 | 77.37 |
| *Bolinus brandaris* | Gastropoda | 15,380 | | 67.26 | 2.07 | 29 | 8 | 3726 | 66.43 | 955 | 68.17 | 1388 | 71.76 |
| *Cancellaria cancellata* | Gastropoda | 16,648 | | 70.15 | 8.81 | 29 | 8 | 3734 | 68.72 | 1087 | 69.37 | 1354 | 72.08 |
| *Cepaea nemoralis* | Gastropoda | 14,100 | | 59.79 | 1.55 | 24 | 13 | 3548 | 58.98 | 710 | 61.55 | 1215 | 61.89 |
| *Chaetoderma nitidulum* | Caudofoveata | 21,008 | | 67.96 | 27.00 | 20 | 18 | 3907 | 65.75 | 730 | 70.00 | 1362 | 70.93 |
| *Chlamys farreri* | Bivalvia | 20,889 | | 58.74 | 29.45 | 34 | 0 | 3736 | 58.91 | 953 | 52.36 | 1479 | 58.28 |
| *Conus textile* | Gastropoda | 15,562 | | 65.19 | 3.40 | 29 | 8 | 3741 | 64.65 | 958 | 65.24 | 1378 | 67.92 |
| *Crassostrea gigas* | Bivalvia | 18,224 | | 63.35 | 11.68 | 39 | 0 | 3648 | 62.90 | 1037+1205 | 60.08 | 1315 | 65.10 |
| *Cristaria plicata* | Bivalvia | 15,712 | | 63.76 | 6.98 | 25 | 11 | 3701 | 62.97 | 844 | 64.22 | 1285 | 64.51 |
| *Cymatium parthenopeum* | Gastropoda | 15,270 | | 69.11 | 1.24 | 29 | 8 | 3733 | 68.44 | 964 | 69.29 | 1360 | 73.60 |
| *Cymbium olla* | Gastropoda | 15,375 | | 68.57 | 2.59 | 30 | 7 | 3733 | 68.00 | 948 | 69.30 | 1348 | 71.44 |
| *Dendropoma gregarium* | Gastropoda | 15,641 | | 60.39 | 3.52 | 30 | 8 | 3706 | 59.78 | 979 | 63.33 | 1349 | 63.53 |
| *Dosidicus gigas* | Cephalopoda | 20,324 | | 69.32 | 6.58 | 21 | 22 | 4736 | 68.09 | 979 | 72.22 | 1424 | 73.24 |
| *Elysia chlorotica* | Gastropoda | 14,132 | | 63.91 | 1.05 | 23 | 13 | 3624 | 63.32 | 713 | 66.06 | 1037 | 66.73 |
| *Fusiturris similis* | Gastropoda | 15,595 | | 66.37 | 3.58 | 29 | 8 | 3735 | 64.86 | 944 | 68.11 | 1365 | 72.89 |
| *Graptacme eborea* | Scaphopoda | 14,492 | | 74.12 | 0.79 | 19 | 18 | 3653 | 73.33 | 729 | 74.18 | 1241 | 76.35 |
| *Haliotis rubra* | Gastropoda | 16,907 | | 59.11 | 9.35 | 15 | 22 | 3738 | 57.55 | 1067 | 59.51 | 1491 | 62.78 |
| *Hiatella arctica* | Bivalvia | 18,244 | | 66.35 | 13.62 | 38 | 0 | 3968 | 65.87 | 901 | 63.04 | 1447 | 66.97 |
| *Hyriopsis schlegelii* F | Bivalvia | 15,939 | | 60.30 | 8.12 | 26 | 11 | 3690 | 59.92 | 835 | 60.00 | 1295 | 60.39 |
| *Katharina tunicata* | Polyplacophora | 15,532 | | 69.47 | 5.04 | 16 | 21 | 3721 | 67.68 | 826 | 72.24 | 1275 | 74.04 |
| *Lampsilis ornata* | Bivalvia | 16,060 | | 62.41 | 8.04 | 26 | 11 | 3710 | 61.67 | 846 | 60.52 | 1315 | 63.10 |
| *Lasmigona compressa* | Bivalvia | 15,903 | | 66.88 | 4.23 | 27 | 10 | 3711 | 67.10 | 848 | 63.09 | 1275 | 65.96 |
| *Loligo bleekeri* | Cephalopoda | 17,211 | | 71.27 | 14.28 | 16 | 21 | 3725 | 69.98 | 782 | 76.21 | 1302 | 74.42 |
| *Loripes lacteus* | Bivalvia | 17,321 | | 62.14 | 14.56 | 37 | 0 | 3751 | 60.50 | 840 | 64.76 | 1187 | 66.05 |
| *Lucinella divaricata* | Bivalvia | 18,940 | | 63.72 | 21.65 | 37 | 0 | 3765 | 61.61 | 836 | 64.47 | 1185 | 66.92 |
| *Margaritifera falcata* | Bivalvia | 16,121 | | 61.69 | 5.87 | 27 | 10 | 3706 | 61.12 | 855 | 60.00 | 1326 | 62.14 |
| *Meretrix petechialis* | Bivalvia | 19,567 | | 68.32 | 16.68 | 36 | 0 | 4014 | 66.93 | 1187 | 69.42 | 1581 | 71.03 |
| *Micromelo undata* | Gastropoda | 14,160 | | 66.29 | 0.60 | 24 | 13 | 3604 | 65.88 | 723 | 63.90 | 1068 | 69.38 |
| *Mimachlamys nobilis* | Bivalvia | 17,963 | | 59.33 | 19.00 | 34 | 0 | 3610 | 60.19 | 939 | 56.55 | 1395 | 61.15 |
| *Mizuhopecten yessoensis* | Bivalvia | 20,414 | | 55.20 | 41.89 | 28 | 0 | 3742 | 55.61 | 961 | 50.57 | 1424 | 57.79 |
| *Musculista senhousia* F | Bivalvia | 21,557 | | 66.54 | 32.08 | 36 | 0 | 3738 | 65.04 | 819 | 69.35 | 1125 | 66.22 |
| *Myosotella myosotis* | Gastropoda | 14,246 | | 55.02 | 2.01 | 24 | 13 | 3576 | 54.51 | 712 | 55.90 | 1089 | 57.67 |
| *Mytilus galloprovincialis* F | Bivalvia | 16,744 | | 61.77 | 10.80 | 37 | 0 | 3732 | 60.55 | 947 | 64.20 | 1244 | 65.59 |
| *Nassarius reticulatus* | Gastropoda | 15,271 | | 67.45 | 1.67 | 29 | 8 | 3732 | 66.69 | 961 | 68.26 | 1353 | 71.69 |
| *Nautilus macromphalus* | Cephalopoda | 16,258 | | 59.58 | 9.03 | 14 | 22 | 3711 | 58.19 | 891 | 60.61 | 1348 | 62.54 |
| *Notodoris gardineri* | Gastropoda | 14,424 | | 66.04 | 3.85 | 24 | 13 | 3544 | 65.68 | 713 | 67.32 | 1079 | 67.93 |
| *Onchidella celtica* | Gastropoda | 14,150 | | 59.31 | 0.42 | 26 | 14 | 3577 | 59.12 | 708 | 58.90 | 1056 | 63.73 |
| *Oncomelania hupensis* | Gastropoda | 15,182 | | 67.32 | 1.35 | 29 | 8 | 3729 | 66.37 | 943 | 68.72 | 1339 | 72.07 |
| *Ostrea edulis* | Bivalvia | 16,320 | | 64.86 | 10.50 | 38 | 0 | 3628 | 64.62 | 924 | 60.06 | 1237 | 64.59 |
| *Paphia euglypta* | Bivalvia | 18,643 | | 66.87 | 13.65 | 37 | 0 | 3968 | 66.02 | 1236 | 68.28 | 1433 | 69.99 |
| *Placopecten magellanicus* | Bivalvia | 32,115 | | 55.67 | 50.86 | 46 | 0 | 3742 | 55.73 | 970 | 52.89 | 1387 | 58.04 |
| *Platynereis dumerilii* | Polychaeta | 15,619 | | 64.14 | 8.04 | 37 | 0 | 3661 | 63.19 | 790 | 63.16 | 1172 | 64.33 |
| *Pupa strigosa* | Gastropoda | 14,189 | | 61.13 | 1.16 | 25 | 12 | 3593 | 60.63 | 729 | 62.55 | 1069 | 63.70 |
| *Pyganodon grandis* F | Bivalvia | 15,848 | | 64.27 | 7.21 | 26 | 11 | 3712 | 63.65 | 847 | 60.92 | 1271 | 64.67 |
| *Pyramidella dolabrata* | Gastropoda | 13,856 | | 63.42 | 0.34 | 22 | 15 | 3575 | 62.97 | 695 | 62.34 | 998 | 65.83 |
| *Quadrula quadrula* F | Bivalvia | 16,033 | | 62.62 | 8.41 | 26 | 11 | 3687 | 61.94 | 857 | 61.49 | 1297 | 63.99 |
| *Saccostrea mordax* | Bivalvia | 16,532 | | 64.41 | 10.44 | 36 | 0 | 3678 | 63.73 | 947 | 59.56 | 1374 | 64.41 |
| *Sepia officinalis* | Cephalopoda | 16,163 | | 72.69 | 7.31 | 15 | 22 | 3748 | 71.10 | 984 | 75.30 | 1266 | 76.15 |
| *Sinanodonta woodiana* | Bivalvia | 16,243 | | 65.83 | 7.82 | 26 | 11 | 3700 | 64.61 | 847 | 64.58 | 1284 | 66.74 |
| *Sinonovacula constricta* | Bivalvia | 17,225 | | 67.05 | 13.47 | 33 | 0 | 3825 | 66.55 | 911 | 65.75 | 1231 | 69.46 |
| *Siphonaria pectinata* | Gastropoda | 14,065 | | 66.82 | 1.32 | 24 | 13 | 3592 | 67.36 | 693 | 62.77 | 1022 | 67.22 |
| *Solemya velum* | Bivalvia | 15,660 | | 68.11 | 4.12 | 18 | 19 | 3735 | 67.14 | 885 | 68.93 | 1376 | 70.57 |
| *Sthenoteuthis oualaniensis* | Cephalopoda | 20,306 | | 69.11 | 6.46 | 21 | 22 | 4736 | 67.81 | 983 | 73.55 | 1430 | 72.31 |
| *Terebra dimidiata* | Gastropoda | 16,513 | | 65.65 | 8.71 | 29 | 8 | 3732 | 65.02 | 964 | 63.28 | 1388 | 67.65 |
| *Unio pictorum* | Bivalvia | 15,760 | | 65.15 | 6.41 | 26 | 11 | 3708 | 64.62 | 859 | 62.63 | 1302 | 65.59 |
| *Utterbackia peninsularis* | Bivalvia | 16,803 | | 65.04 | 4.45 | 26 | 11 | 3924 | 65.42 | 840 | 62.02 | 1313 | 62.53 |
| *Venerupis philippinarum* F | Bivalvia | 22,676 | | 69.70 | 25.98 | 37 | 0 | 4211 | 68.56 | 1249 | 70.62 | 1408 | 72.80 |
| *Venustaconcha ellipsiformis* F | Bivalvia | 15,975 | | 62.55 | 7.72 | 26 | 11 | 3713 | 61.81 | 865 | 60.92 | 1271 | 64.20 |
| Sample size | Class/Species |  | Genome features | | | | | PCGs | | *rrnS* | | *rrnL* | |
|  |  | Length  (bp) | | A+T  (%) | %UNs | H | L | aa | A+T  (%) | Length  (bp) | A+T  (%) | Length  (bp) | A+T  (%) |
| (*N* = 29) | Bivalvia | 18,052.35 | | 63.51 | 14.31 | 33.10 | 3.88 | 3785.08 | 62.81 | 939.64 | 62.21 | 1343.40 | 64.98 |
| (*N* = 1) | *Solemya velum* | 15,660.00 | | 68.11 | 4.12 | 18.00 | 19.00 | 3735.00 | 67.14 | 885.00 | 68.93 | 1376.00 | 70.57 |
| (*N* = 1) | Caudofoveata | 21,008.00 | | 67.96 | 27.00 | 20.00 | 18.00 | 3907.00 | 65.75 | 730.00 | 70.00 | 1362.00 | 70.93 |
| (*N* = 6) | Cephalopoda | 18,432.17 | | 68.62 | 8.44 | 18.00 | 21.83 | 4231.50 | 67.26 | 922.50 | 71.86 | 1362.83 | 72.02 |
| (*N* = 23) | Gastropoda | 15,027.58 | | 64.91 | 3.18 | 25.46 | 11.67 | 3657.17 | 64.30 | 850.96 | 65.06 | 1223.79 | 67.67 |
| (*N* = 1) | Polychaeta | 15,619.00 | | 64.14 | 8.04 | 37.00 | 0.00 | 3661.00 | 63.19 | 790.00 | 63.16 | 1172.00 | 64.33 |
| (*N* = 1) | Polyplacophora | 15,532.00 | | 69.47 | 5.04 | 16.00 | 21.00 | 3721.00 | 67.68 | 826.00 | 72.24 | 1275.00 | 74.04 |
| (*N* = 1) | Scaphopoda | 14,492.00 | | 74.12 | 0.79 | 19.00 | 18.00 | 3653.00 | 73.33 | 729.00 | 74.18 | 1241.00 | 76.35 |
