# Supplementary figures and images for: The complete mitochondrial genome of Solemya velum (Mollusca: Bivalvia) and its relationships with Conchifera
Source: BMC Genomics. 2013 Jun 18;14:409. doi: 10.1186/1471-2164-14-409 (PMC3704766; doi:10.1186/1471-2164-14-409)

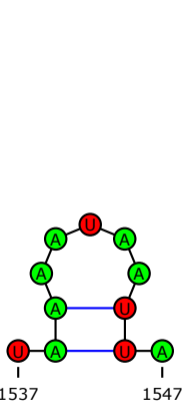

*cox1-cox2*

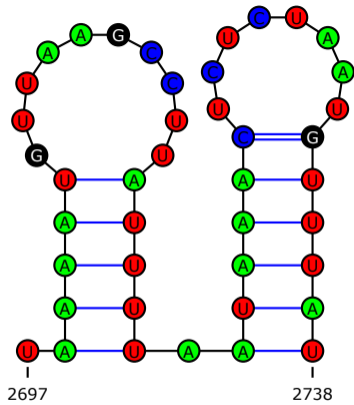

*nad4L-nad4*

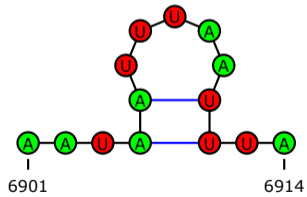

*atp8-cytb*

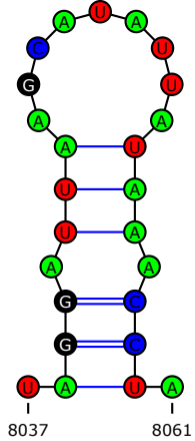

*cytb-nad6*

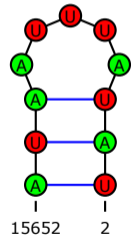

*nad2-cox1*

Supplement: Additional file 7 — Secondary structures of regions between two consecutive PCGs. [file 1471-2164-14-409-S7.pdf]

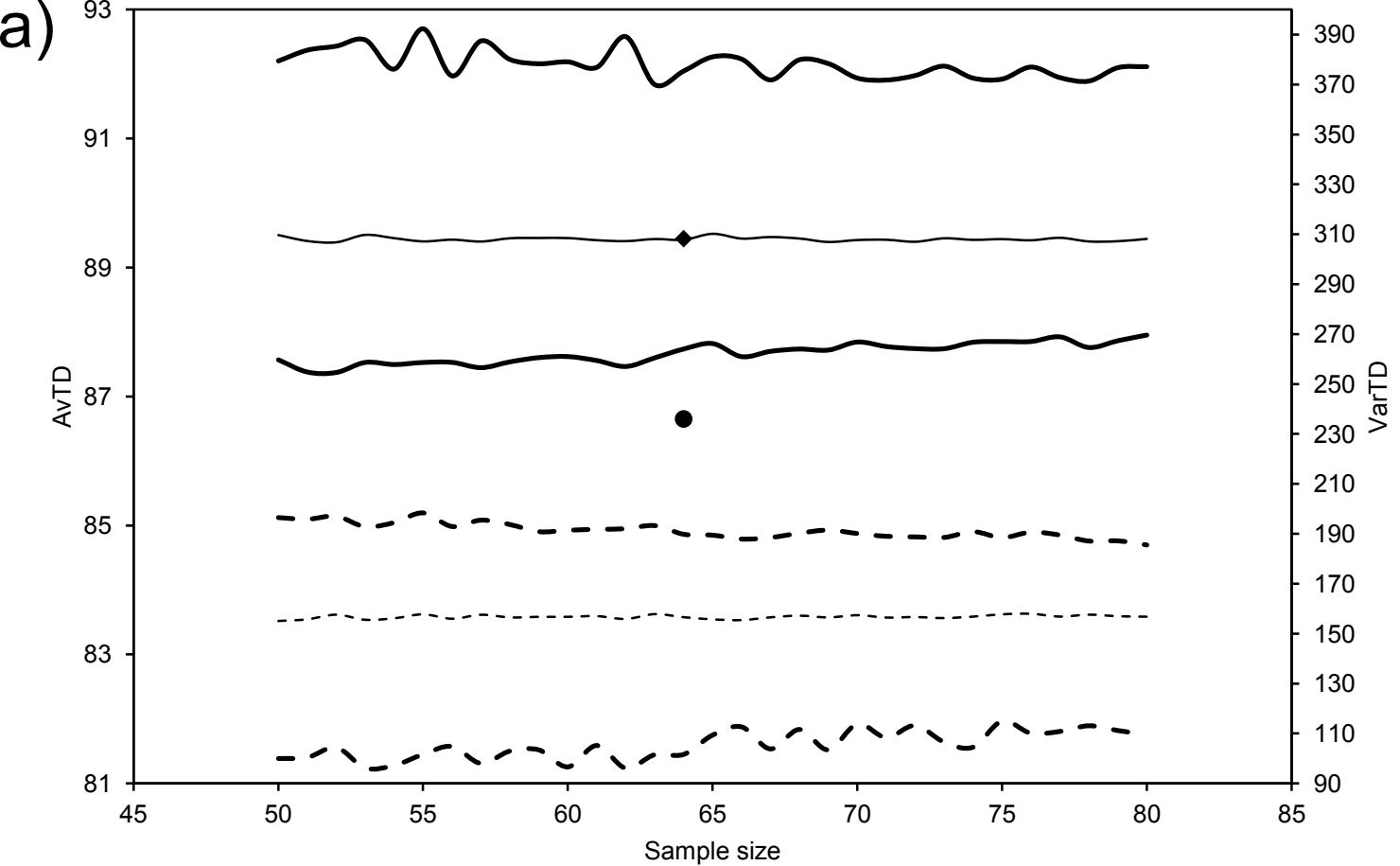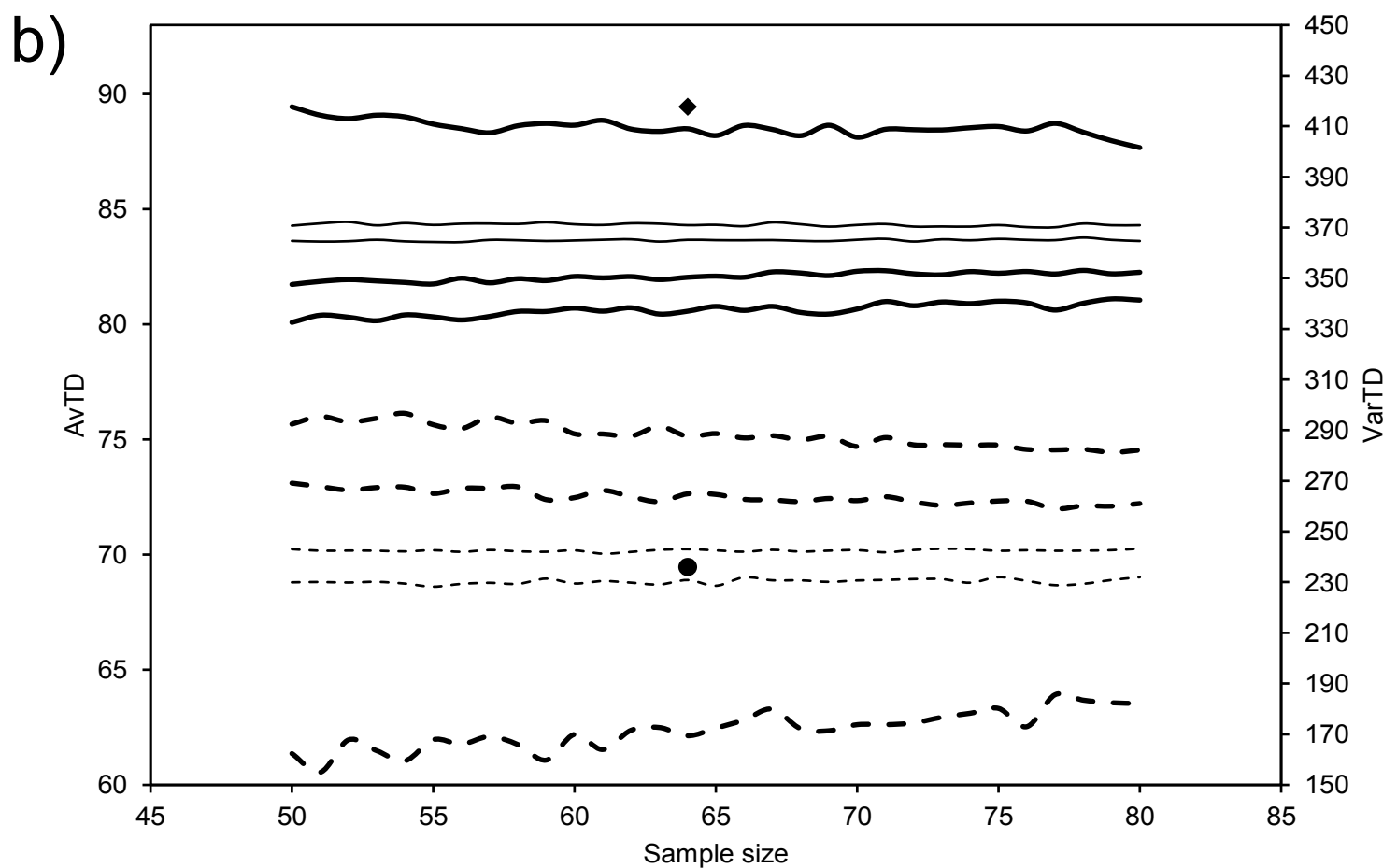

Supplement: Additional file 10 — Phylogenetic Representativeness. Test is reported as in [49] for (a) the original master list of mollusks taken from [93] and (b) a set of 100 shuffled master lists simulating taxonomical revisions. In both cases, AvTD is plotted on the left axis in the upper part of the chart and VarTD on the right one in the lower part. Sample size is plotted on x-axis; the greatest AvTD value (upper thick continue line), the AvTD mean (thin continue line), the AvTD 95% lower confidence limit (lower thick continue line), the VarTD 95% upper confidence limit (upper thick dashed line), the VarTD mean (thin dashed line), and the lowest VarTD value (lower thick dashed line) are shown. All these lines (with the exception of greatest AvTD and lowest VarTD) are shown as two-tailed 95% confidence limits for shuffling test (b). Sample used for this work is shown as a black diamond (AvTD)/circle (VarTD). [file 1471-2164-14-409-S10.pdf]
