# Supplementary material for: The complete mitochondrial genome of Solemya velum (Mollusca: Bivalvia) and its relationships with Conchifera
Source: BMC Genomics. 2013 Jun 18;14:409. doi: 10.1186/1471-2164-14-409 (PMC3704766; doi:10.1186/1471-2164-14-409)
Supplement: Additional file 8 — Codon usage in Solemya velum mitochondrial genome. The total frequency of each amminoacid is reported under the three-letter/one-letter name; underlined codons correspond to anticodons of mitochondrial tRNAs. All truncated (TA-/T--) stop codons were attributed to TAA. RSCU, Relative Synonymous Codon Usage. [file 1471-2164-14-409-S8.doc]

| aa | Codon | Count | % | RSCU | aa | Codon | Count | % | RSCU | aa | Codon | Count | % | RSCU | aa | Codon | Count | % | RSCU |
| --- | --- | --- | --- | --- | --- | --- | --- | --- | --- | --- | --- | --- | --- | --- | --- | --- | --- | --- | --- |
| Phe (F) | UUU | 247 | 6.59 | 1.57 | Ser (S) | UCU | 106 | 2.83 | 2.15 | Ala (A) | GCU | 87 | 2.32 | 1.57 | Asp (D) | GAU | 37 | 0.99 | 1.03 |
| 8.40% | UUC | 68 | 1.81 | 0.43 | 10.54% | UCC | 27 | 0.72 | 0.55 | 5.90% | GCC | 46 | 1.23 | 0.83 | 1.92% | GAC | 35 | 0.93 | 0.97 |
| Leu (L) | UUA | 269 | 7.18 | 2.92 |  | UCA | 102 | 2.72 | 2.07 |  | GCA | 74 | 1.97 | 1.34 | Glu (E) | GAA | 59 | 1.57 | 1.37 |
| 14.73% | UUG | 50 | 1.33 | 0.54 |  | UCG | 11 | 0.29 | 0.22 |  | GCG | 14 | 0.37 | 0.25 | 2.29% | GAG | 27 | 0.72 | 0.63 |
|  | CUU | 91 | 2.43 | 0.99 |  | AGU | 32 | 0.85 | 0.65 | Tyr (Y) | UAU | 84 | 2.24 | 1.22 | Cys (C) | UGU | 31 | 0.83 | 1.44 |
|  | CUC | 24 | 0.64 | 0.26 |  | AGC | 17 | 0.45 | 0.34 | 3.68% | UAC | 54 | 1.44 | 0.78 | 1.15% | UGC | 12 | 0.32 | 0.56 |
|  | CUA | 104 | 2.77 | 1.13 |  | AGA | 68 | 1.81 | 1.38 | Stop (*) | UAA | 11 | 0.29 | 1.69 | Trp (W) | UGA | 85 | 2.27 | 1.52 |
|  | CUG | 14 | 0.37 | 0.15 |  | AGG | 32 | 0.85 | 0.65 | 0.35% | UAG | 2 | 0.05 | 0.31 | 2.99% | UGG | 27 | 0.72 | 0.48 |
| Ile (I) | AUU | 267 | 7.12 | 1.72 | Pro (P) | CCU | 71 | 1.89 | 1.97 | His (H) | CAU | 45 | 1.20 | 1.20 | Arg (R) | CGU | 12 | 0.32 | 0.76 |
| 8.27% | AUC | 43 | 1.15 | 0.28 | 3.84% | CCC | 11 | 0.29 | 0.31 | 2.00% | CAC | 30 | 0.80 | 0.80 | 1.68% | CGC | 2 | 0.05 | 0.13 |
| Met (M) | AUA | 202 | 5.39 | 1.58 |  | CCA | 43 | 1.15 | 1.19 | Gln (Q) | CAA | 51 | 1.36 | 1.46 |  | CGA | 40 | 1.07 | 2.54 |
| 6.83% | AUG | 54 | 1.44 | 0.42 |  | CCG | 19 | 0.51 | 0.53 | 1.87% | CAG | 19 | 0.51 | 0.54 |  | CGG | 9 | 0.24 | 0.57 |
| Val (V) | GUU | 110 | 2.93 | 1.83 | Thr (T) | ACU | 75 | 2.00 | 1.64 | Asn (N) | AAU | 94 | 2.51 | 1.46 | Gly (G) | GGU | 46 | 1.23 | 0.77 |
| 6.43% | GUC | 10 | 0.27 | 0.17 | 4.88% | ACC | 17 | 0.45 | 0.37 | 3.44% | AAC | 35 | 0.93 | 0.54 | 6.35% | GGC | 33 | 0.88 | 0.55 |
|  | GUA | 93 | 2.48 | 1.54 |  | ACA | 81 | 2.16 | 1.77 | Lysine (K) | AAA | 76 | 2.03 | 1.65 |  | GGA | 103 | 2.75 | 1.73 |
|  | GUG | 28 | 0.75 | 0.46 |  | ACG | 10 | 0.27 | 0.22 | 2.45% | AAG | 16 | 0.43 | 0.35 |  | GGG | 56 | 1.49 | 0.94 |
