# Supplementary material for: The complete mitochondrial genome of Solemya velum (Mollusca: Bivalvia) and its relationships with Conchifera
Source: BMC Genomics. 2013 Jun 18;14:409. doi: 10.1186/1471-2164-14-409 (PMC3704766; doi:10.1186/1471-2164-14-409)
Supplement: Additional file 9 — Unassigned regions longer than 10 bp. [file 1471-2164-14-409-S9.doc]

| UR ID | Start | Stop | Length (bp) |
| --- | --- | --- | --- |
| UR1 | 1537 | 1553 | 17 |
| UR2 | 2241 | 2271 | 31 |
| UR3 | 2708 | 2718 | 11 |
| UR4 | 5928 | 5958 | 31 |
| UR5 | 6901 | 6914 | 14 |
| UR6 | 8038 | 8058 | 21 |
| UR7 | 12463 | 12567 | 105 |
| UR8 | 13487 | 13858 | 372 |
