# Supplementary material for: The complete mitochondrial genome of Solemya velum (Mollusca: Bivalvia) and its relationships with Conchifera
Source: BMC Genomics. 2013 Jun 18;14:409. doi: 10.1186/1471-2164-14-409 (PMC3704766; doi:10.1186/1471-2164-14-409)
Supplement: Additional file 11 — Amminoacid substitution models selected by ProtTest 3.2 [80] and piped to RAxML 7.2.8 [82,83]. [file 1471-2164-14-409-S11.doc]

| Partition | Model |
| --- | --- |
| concatenated alignment | LGF |
| *atp6*-*cytb*-*nad2*-*nad3*-*nad4*-*nad5* | MTARTF |
| *cox1*-*cox2*-*cox3*-*nad1* | LGF |
| *atp6* | MTART |
| *cox1* | LGF |
| *cox2* | LGF |
| *cox3* | LGF |
| *cytb* | MTARTF |
| *nad1* | MTART |
| *nad2* | MTART |
| *nad3* | MTART |
| *nad4* | MTARTF |
| *nad5* | LGF |
| *nad6* | MTART |
